# Associations between genotype, phenotype and behaviours measured by the Rett syndrome behaviour questionnaire in Rett syndrome

**DOI:** 10.1186/s11689-024-09575-4

**Published:** 2024-10-25

**Authors:** Jenny Downs, Kingsley Wong, Helen Leonard

**Affiliations:** 1 Centre for Child Health Research, The Kids Research Institute Australia, University of Western Australia, 15 Hospital Avenue, Nedlands, WA 6009 Australia; 2 Curtin School of Allied Health, Curtin University, GPO Box U1987, Perth, WA 6845 Australia

**Keywords:** Rett syndrome, Behaviour, Outcome measure, Genotype, Phenotype

## Abstract

**Introduction:**

Rett syndrome (RTT) is a rare neurodevelopmental disorder with developmental impairments, comorbidities, and abnormal behaviours such as hand stereotypies and emotional features. The Rett Syndrome Behaviour Questionnaire (RSBQ) was developed to describe the behavioural and emotional features of RTT. Little is known how RSBQ scores are associated with genetic and clinical characteristics in RTT. This study investigated relationships between genotype, age, walking, hand function, sleep, and RSBQ total and subscale scores in RTT.

**Methods:**

This is a cross-sectional analysis of data collected in the Australian Rett Syndrome Database and the International Rett Syndrome Phenotype Database. Parent caregivers completed the RSBQ and Sleep Disturbance Scale for Children [subscales for disorders of initiating and maintaining sleep (DIMS), disorders of excessive somnolence (DOES)], and provided information on age, variant type, functional abilities (mobility, hand function), seizure frequency and gastrointestinal problems. Associations between the RSBQ scores and the independent variables were modelled using linear regression.

**Results:**

Data were available for 365 individuals with RTT [median (range) age 17.8 (2.9–51.9) years, 2 males]. Compared to adults, 2- to 12-year-old children had higher mean Total, Night-time Behaviour and Fear/Anxiety scores. Compared to individuals with a C-terminal deletion, individuals with the p.Arg255* variant had higher mean Total and Night-time Behaviours scores, whereas the p.Arg294* variant had higher mean Mood scores. Individuals with intermediate mobility and hand function abilities had a higher mean Total score. Total RSBQ and subscale scores were similar across categories for seizures, constipation, and reflux, but were higher with abnormal DIMS and abnormal DOES scores.

**Conclusion:**

Except for associations with sleep, the RSBQ measures the behavioural phenotype rather than clinical severity in RTT, as traditionally conceptualised in terms of functional abilities and comorbidities. When designing clinical trials, the RSBQ needs to be complemented by other outcome measures to assess specific core functions and associated comorbidities in RTT.

**Supplementary Information:**

The online version contains supplementary material available at 10.1186/s11689-024-09575-4.

## Introduction

Rett syndrome (RTT) is a severe neurodevelopmental disorder, caused by a pathogenic variant on the X-linked methyl CpG binding protein 2 (*MECP2)* gene [1], affecting approximately 1 in 9000 liveborn females [2]. The core symptoms include a history of regression of hand and communication skills accompanied by the development of hand stereotypies and gait abnormality, with ongoing impacts on hand, communication and gross motor functions [3]. The disorder is also associated with multiple comorbidities including scoliosis, autonomic dysfunction, epilepsy, poor growth and sleep difficulties [3, 4]. There are relationships between genotype and clinical presentation. For example, variants such as Large Deletions, p.Arg555* and p.Arg270* are usually severe whereas C-terminal deletions, p.Arg133Cys, p.Arg306Cys and p.Arg294* are generally milder [4]. 

Beyond the core diagnostic criteria, there are also abnormal behaviours and emotional features such as fluctuations in mood or screaming at night. The Rett Syndrome Behaviour Questionnaire (RSBQ) was developed more than 20 years ago prior to the discovery of the genetic cause for RTT to differentiate the specific behavioural symptoms of female children with RTT from those of females with other severe intellectual disability [5]. Including data derived from an earlier caregiver survey for 143 females with RTT [6], a principal component analysis of items was conducted yielding a 45-item and 8-domain questionnaire for completion by caregivers [5]. There were moderate to high values for internal consistency and good inter-rater and test-retest reliability [5]. The RSBQ was the first behavioural measure developed specifically for RTT.

The use of the RSBQ was subsequently expanded to adults [7, 8], and in the absence of other measures for RTT, it has been approved as an outcome measures for recent clinical trials [9–12]. An Australian study of females with RTT aged from 3 to 27 years (*n* = 145) found that some variants that were usually associated with milder symptoms had higher RSBQ scores [8]. For example, higher Fear/Anxiety scores were more common in individuals with the p.Arg133Cys and pArg306Cys variants and individuals with the p.Arg294* variant were more likely to have high General Mood and Body Rocking scores although low Hand Behaviours and Face Movements scores [8]. In a UK study of females with RTT aged from 4 to 47 years, it was reported that RSBQ scores did not vary with age and in a subset of 50 individuals they appeared stable after a 16-month interval [7]. In a subsequent study undertaken at Boston Children’s Hospital in 2015 with 74 females aged 2 to 11 years, the RSBQ was one of three tools evaluated to measure anxiety in RTT [13]. The Anxiety, Depression and Mood Scale was found to have the best psychometric properties for RTT, and again higher Fear/Anxiety subscale scores from the RSBQ were observed in individuals with milder clinical severity [13]. 

In clinical trials, the Fear/Anxiety subscale from the RSBQ was a primary endpoint for the mecasermin (recombinant human insulin-like growth factor 1, rhIGF-1) trial and scores were similar for the treatment and placebo groups [10]. However, in the later LAVENDER clinical trial, a 12-week, multi-center, randomized, double-blind, placebo-controlled, Phase 3 study of trofinetide efficacy in 187 females with RTT, there was a statistical significant between-group difference in favour of trofinetide (mean change − 3.1, 95% confidence interval − 5.7, -0.6; *P* = 0.0175; Cohen’s *d* effect size = 0.37) for the RSBQ Total score, based on the mixed-effect model for repeated measure analysis [14]. The trofinetide group also saw a notable improvement in the Fear/Anxiety, Body rocking and expressionless face subscale scores compared to the placebo group, although the study was not powered to detect subscale differences [14]. 

Use of the RSBQ in the mecasermin and trofinetide clinical trials has been the catalyst for new validation studies. In a recent US study, 149 caregivers of 2 to 33-year-old individuals with RTT completed the RSBQ at baseline, after 1–2 weeks and again after 1-2months [15]. Analyses found that more than half of caregivers rated 18 items as 0 (“not a problem”) and 6 items as 2 (often a problem) suggesting potential floor or ceiling effects for those items, item agreement between testing occasions was mostly moderate, and test-retest reliability was poor [15]. A recent factor analysis of the RSBQ using the largest sample size (323 children, 309 adults) to date involved multiple sources in the US, UK, Denmark and Australia [16] The 8-factor structure was generally replicated but, a 6-factor solution and a 7-factor solution were also found for children and adults respectively. A new factor combined the original General Mood and Night-time Behaviours subscales and was called Emotional and Disruptive Behaviours (12 items) [16]. 

There remains little information on how RSBQ scores are associated with genetic and clinical characteristics and how scores can be interpreted in upcoming surveillance studies and clinical trials. Therefore, the aim of this study was to update and expand previous investigations on relationships between age, genotype, movement abnormalities (hand function/walking ability), sleep and RSBQ Total and subscale scores in RTT.

## Methods

### Data sources

There were two data sources for this study. The Australian Rett Syndrome Database (AussieRett) was established in 1993 and is population-based and longitudinal with multiple waves of data collection [17]. The 2019 follow-up questionnaire was administered to English-speaking parent caregivers of 177 individuals with a confirmed *MECP2* variant using the REDCap platform [18] and completed questionnaires were returned by 148 (83.6%). The International Rett Syndrome Phenotype Database (InterRett) was established in 2002 [19] and follow up questionnaires were administered in 2015 [20] and 2018 [21], also administered using the REDCap platform [18]. Parents are invited to provide a copy of their child’s genetic report when registering with the database. The 2018 follow-up questionnaires were administered to English-speaking parent caregivers of 233 individuals with a confirmed *MECP2* variant and completed questionnaires were returned by 216 (92.7%).

### Outcome measure

The RSBQ [5] was included in the most recent questionnaire (2019 for AussieRett, 2018 for InterRett) for both data sources. The RSBQ comprises 45 items which are rated on a three-point Likert scale. Items group into eight subscales describing General Mood (8 items), Breathing Problems (5 items), Hand Behaviours (6 items), Face Movements (4 items), Body Rocking (6 items), Night-time Behaviours (3 items), Fear/Anxiety (4 items) and Walking/Standing (2 items). Forty-five items contribute to the Total Score where the total possible score is 90 [5]. The new factor that combined the original General Mood and Night-time Behaviours subscales and was called Emotional and Disruptive Behaviours (12 items) was included in the analysis [16]. 

### Independent variables

Current age was categorised as ‘2–12 years’, ’13–17 years’, ‘18–24 years’, or ’25 years and older’. Genetic variants were grouped as C-terminal deletion, early truncating, large deletion, p.Arg106Trp, p.Arg133Cys, p.Thr158Met, p.Arg168*, p.Arg255*, p.Arg270*, p.Arg294*, p.Arg306Cys, and all other pathogenic mutations were grouped as ‘other’. Parents reported current functional abilities and health status. Mobility (walking) was categorised as follows: ‘unable’, ‘assisted’, and ‘independent’. Hand function was classified as ‘unable’, ‘able to pick up large objects’, and ‘able to pick up small objects’. Seizure frequency over the previous 12 months was categorised as: ‘never or controlled’, ‘monthly or less’, ‘weekly’, or ‘daily’. Constipation and gastro-esophageal reflux were coded as present at the time of questionnaire completion or not. The Sleep Disorder Scale for Children (SDSC) is a validated measure for reporting sleep problems in children [22]. The SDSC comprises 26 items that are rated on a five-point Likert scale and grouped into six subscales. The disorders of initiating and maintaining sleep (DIMS) and the disorders of excessive somnolence (DOES) subscales were used for the current study. Each subscale was scored through the summation of all the subscale items. The scores were then compared with the normative data reported in the initial validation paper [22] as follows. Each score was subtracted from the mean subscale score divided by the standard deviation of the normative DIMS or DOES dataset to calculate a z-score. The z-score was then transformed to a t-score by multiplying by 10 and adding 50. The t-score was dichotomised as: scores within normal range (“below 70”) and scores outside of normal range (“70 and above”).

The Child and Adolescent Health Services Human Research Ethics Committee approved the AussieRett study (RGS2390) and the University of Western Australia Human Research Ethics Committee approved the InterRett study (2021/ET000616). Primary caregivers provided informed written consent to participate.

### Statistical analysis

Descriptive statistics were used to summarise the characteristics of the study population. Categorical data were summarised using frequency and proportion, and continuous data such as the outcome measures were summarised using mean and standard deviation. The associations between the RSBQ subscale and total scores and the independent variables were modelled using simple and multiple linear regression and the unadjusted and adjusted coefficients (and their 95% confidence intervals) were subsequently estimated, respectively. All covariates were included in the multiple regression models. Missing RSBQ subscale data (0.2–3.3% by subscale, 0.9% overall) and missing non-subscale items data (1.8%) were imputed using the median score of the completed items provided less than half of the subscale or non-subscale item data was missing. Non-subscale items were treated as a separate group and imputation was carried out the same as subscale items. Missing values in the independent variables were sparse and thus the complete case analysis method was used in the regression analysis. All analyses were performed using Stata version 18.0 (StatCorp, College Station, TX, USA).

## Results

Data were available for 365 individuals with RTT (2 males) and are presented in Table 1. The median age (range) was 17.8 (2.9–51.9) years. Each of the common variant groups were represented. Approximately one third could walk independently and 40.3% could pick up small objects. Comorbidities were common including seizures (57.8%), constipation (30.1%), reflux (40.3%), insomnia (28.5%) and excessive daytime sleepiness (23.6%). There was a larger proportion of 2–12 year old children in AussieRett than in the InterRett sample (32.4% vs. 19.8%); a smaller proportion of individuals with the p.Arg255* variant in AussieRett than in the InterRett sample (5.4% vs. 13.8%); a larger proportion in the AussieRett sample had constipation compared with the InterRett sample (54.1% vs. 13.8%), and a smaller proportion in the AussieRett sample had reflux compared with the InterRett sample (20.3% vs. 53.9%). See Table 1.

**Table 1:** Characteristics of the children and adults with Rett syndrome

	AussieRett	InterRett	Overall
N	148	217	365
Age, in years			
Mean (SD)	18.1 (9.4),	20.4 (9.6),	19.5 (9.5),
Median range (IQR)	17.2 (10.5–24.3),	18.0 (13.7–25.3),	17.8 (13.0-24.7),
Range	2.9–40.1	6.2–51.9	2.9–51.9
2–12	48 (32.4)	43 (19.8)	91 (24.9)
13–17	30 (20.3)	65 (30.0)	95 (26.0)
18–24	36 (24.3)	53 (24.4)	89 (24.4)
25+	34 (23.0)	56 (25.8)	90 (24.7)
Variant, n (%)			
C-terminal deletion	21 (14.2)	18 (8.3)	39 (10.7)
Early truncation	12 (8.1)	14 (6.5)	26 (7.1)
Large deletion	14 (9.5)	14 (6.5)	28 (7.7)
p.Arg106Trp	7 (4.7)	11 (5.1)	18 (4.9)
p.Arg133Cys	13 (8.8)	16 (7.4)	29 (7.9)
p.Thr158Met	13 (8.8)	21 (9.7)	34 (9.3)
p.Arg168*	14 (9.5)	26 (12.0)	40 (11.0)
p.Arg255*	8 (5.4)	30 (13.8)	38 (10.4)
p.Arg270*	12 (8.1)	10 (4.6)	22 (6.0)
p.Arg294*	7 (4.7)	14 (6.5)	21 (5.8)
Arg306Cys	10 (6.8)	12 (5.5)	22 (6.0)
Other	13 (8.8)	22 (10.1)	35 (9.6)
Unknown	4 (2.7)	9 (4.1)	13 (3.6)
Walking ability, n (%)			
Unable	50 (33.8)	74 (34.1)	124 (34.0)
Assisted	49 (33.1)	67 (30.9)	116 (31.8)
Independent	48 (32.4)	74 (34.1)	122 (33.4)
Missing	1 (0.7)	2 (0.9)	3 (0.8)
Hand function, n (%)			
Unable	42 (28.4)	95 (43.8)	137 (37.5)
Large objects	24 (16.2)	57 (26.3)	81 (22.2)
Small objects	82 (55.4)	65 (30.0)	147 (40.3)
Seizure frequency, n (%)			
Never or controlled	58 (39.2)	96 (44.2)	154 (42.2)
Monthly or less	45 (30.4)	65 (30.0)	110 (30.1)
Weekly	27 (18.2)	28 (12.9)	55 (15.1)
Daily	18 (12.2)	27 (12.4)	45 (12.3)
Missing	-	1 (0.5)	1 (0.3)
Constipation, n (%)			
No	68 (45.9)	186 (85.7)	254 (69.6)
Yes	80 (54.1)	30 (13.8)	110 (30.1)
Missing	-	1 (0.5)	1 (0.3)
GE-Reflux, n (%)			
No	118 (79.7)	98 (45.2)	216 (59.2)
Yes	30 (20.3)	117 (53.9)	147 (40.3)
Missing	-	2 (0.9)	2 (0.5)
Insomnia**, n (%)			
Normal	100 (67.6)	161 (74.2)	261 (71.5)
Abnormal	48 (32.4)	56 (25.8)	104 (28.5)
Excessive daytime sleepiness**, n (%)			
Normal	99 (66.9)	180 (82.9)	279 (76.4)
Abnormal	49 (33.1)	37 (17.1)	86 (23.6)

** DIMS and DOES (< 70 abnormal, ≥ 70 normal)

RSBQ subscale and total scores are presented in Table 2. The overall mean (SD) Total score was 35 (14). There was little variation by the descriptive categories, except individuals with intermediate walking and hand function abilities had slightly higher scores and the highest scores (> 40) were observed for individuals with abnormal DIMS (insomnia) and DOES (excessive daytime sleepiness) scores. This pattern was generally replicated for each of the subscales, except individuals with the p.Arg294* variant had higher General Mood and lower Face Movements scores, and individuals with the p.Arg306Cys had higher Fear/Anxiety scores. RSBQ scores for the paediatric and adult subgroups are presented in Supplementary Tables 1 and 2 in Additional File 1 and the Total scores were slightly lower for adults (36.6 vs. 33.4).

**Table 2:** Mean RSBQ subscale and total scores by covariates among individuals with Rett syndrome

	Subscale								Total* (0–90)
Factor (range)	Factor 1 General Mood (0–16)	Factor 2 Breathing Problems (0–10)	Factor 3 Hand Behaviours (0–12)	Factor 4 Face Movements (0–8)	Factor 5 Body Rocking (0–12)	Factor 6 Night-time Behaviours (0–6)	Factor 7 Fear/Anxiety (0–8)	Factor 8* Walking/ Standing (0–4)	
	mean (SD)								
Overall	5.4 (3.9)	3.8 (3.0)	7.2 (3.0)	2.4 (1.9)	4.4 (2.1)	1.2 (1.4)	3.3 (2.0)	1.8 (1.5)	35.0 (14.0)
Age, in years									
2–12	6.4 (3.7)	4.3 (2.8)	6.8 (2.9)	3.1 (2.1)	4.5 (2.3)	1.6 (1.5)	3.7 (2.1)	1.7 (1.6)	38.5 (14.7)
13–17	4.9 (3.8)	3.9 (3.3)	7.3 (3.0)	2.4 (2.0)	4.4 (2.0)	0.9 (1.2)	3.4 (1.9)	1.8 (1.6)	34.7 (15.0)
18–24	5.4 (3.9)	3.6 (2.8)	7.5 (2.8)	2.2 (1.7)	4.5 (2.2)	1.4 (1.6)	3.3 (2.1)	1.8 (1.5)	34.5 (13.1)
25+	5.1 (4.0)	3.4 (2.9)	7.2 (3.1)	2.0 (1.7)	4.2 (2.0)	1.0 (1.4)	2.8 (1.8)	1.9 (1.5)	32.4 (12.5)
Variant									
C-terminal deletion	4.6 (3.3)	3.8 (3.1)	6.7 (3.3)	2.2 (1.6)	4.2 (2.1)	1.0 (1.3)	3.2 (2.3)	1.5 (1.4)	32.0 (13.1)
Early truncation	4.9 (3.8)	4.2 (3.3)	7.7 (3.1)	2.4 (2.0)	4.1 (2.0)	1.1 (1.6)	2.6 (2.1)	1.9 (1.6)	34.1 (14.9)
Large deletion	5.4 (3.6)	3.6 (3.3)	6.6 (3.2)	2.6 (1.8)	4.6 (1.7)	1.2 (1.4)	2.9 (1.8)	2.0 (1.5)	34.8 (13.6)
p.Arg106Trp	5.7 (4.4)	3.6 (3.0)	7.7 (3.1)	1.9 (1.1)	5.2 (2.7)	1.2 (1.3)	2.9 (1.7)	2.0 (1.3)	35.3 (14.3)
p.Arg133Cys	5.6 (4.2)	3.1 (2.8)	6.1 (3.3)	2.4 (2.3)	4.4 (2.2)	1.0 (1.3)	3.2 (1.9)	1.9 (1.4)	33.2 (15.5)
p.Thr158Met	5.6 (3.9)	4.0 (3.1)	7.7 (2.6)	2.4 (1.9)	4.3 (1.9)	1.2 (1.4)	3.4 (2.2)	2.1 (1.6)	35.9 (13.7)
p.Arg168*	4.8 (3.2)	3.7 (3.2)	7.9 (2.8)	2.9 (2.1)	4.0 (2.2)	1.5 (1.5)	3.5 (2.2)	1.3 (1.6)	35.7 (14.6)
p.Arg255*	5.1 (4.5)	4.5 (3.3)	7.2 (3.2)	2.7 (2.3)	4.7 (2.2)	1.3 (1.5)	3.4 (2.2)	1.8 (1.7)	36.4 (16.4)
p.Arg270*	5.4 (3.9)	3.9 (2.8)	8.2 (2.4)	2.7 (2.3)	4.3 (2.2)	1.5 (1.6)	3.1 (1.8)	1.7 (1.8)	36.9 (15.6)
p.Arg294*	7.3 (3.7)	3.2 (2.9)	7.0 (2.9)	1.8 (1.8)	4.3 (2.2)	1.6 (1.9)	3.7 (1.7)	1.6 (1.2)	36.1 (11.8)
Arg306Cys	6.4 (3.4)	3.0 (2.3)	7.0 (2.9)	2.4 (1.7)	4.6 (2.0)	1.7 (1.6)	4.0 (2.2)	1.8 (1.4)	34.7 (13.8)
Other	5.9 (4.3)	4.3 (2.3)	7.5 (2.6)	2.3 (1.8)	4.1 (2.4)	1.0 (1.3)	3.1 (1.9)	2.0 (1.7)	35.8 (11.4)
Unknown	5.6 (4.3)	4.0 (3.1)	6.0 (2.8)	2.8 (1.7)	4.9 (1.8)	0.8 (1.4)	3.4 (1.8)	2.0 (1.6)	35.5 (14.3)
Walking ability									
Unable	4.8 (3.5)	3.4 (2.8)	6.9 (3.0)	2.5 (1.8)	3.9 (2.0)	1.1 (1.3)	3.1 (2.1)	1.0 (1.3)	32.0 (13.0)
Assisted	5.8 (4.1)	4.3 (3.0)	8.0 (2.8)	2.5 (2.1)	4.7 (2.2)	1.5 (1.6)	3.8 (2.0)	2.4 (1.4)	38.7 (14.4)
Independent	5.7 (3.9)	3.5 (3.0)	6.8 (3.0)	2.3 (1.9)	4.5 (2.1)	1.1 (1.3)	3.0 (2.0)	2.1 (1.5)	34.3 (13.7)
Hand function									
Unable	4.8 (3.6)	4.0 (2.9)	8.0 (2.6)	2.6 (1.9)	4.3 (2.0)	1.1 (1.4)	3.1 (2.0)	1.7 (1.7)	34.7 (12.3)
Large objects	5.8 (4.1)	4.7 (3.1)	8.5 (2.4)	2.8 (2.0)	4.7 (2.4)	1.3 (1.4)	3.8 (2.0)	1.8 (1.5)	39.5 (15.3)
Small objects	5.9 (3.9)	3.1 (2.9)	5.8 (3.0)	2.1 (1.9)	4.3 (2.1)	1.3 (1.5)	3.3 (2.1)	1.9 (1.4)	32.9 (14.3)
Seizure frequency									
Never or controlled	5.7 (4.0)	3.1 (2.9)	6.7 (3.1)	2.3 (1.9)	4.4 (2.2)	1.2 (1.4)	3.2 (2.1)	1.7 (1.5)	33.5 (14.1)
Monthly or less	5.0 (3.7)	4.1 (3.0)	7.6 (2.9)	2.4 (1.9)	4.3 (2.0)	1.0 (1.2)	3.3 (1.9)	1.8 (1.6)	34.7 (13.0)
Weekly	5.5 (3.5)	4.8 (2.7)	7.7 (2.8)	2.8 (2.1)	4.5 (2.5)	1.5 (1.7)	3.5 (2.0)	1.9 (1.7)	38.4 (15.3)
Daily	5.6 (4.1)	4.2 (3.1)	7.5 (2.6)	2.5 (1.7)	4.4 (1.8)	1.6 (1.7)	3.5 (2.1)	2.0 (1.5)	36.8 (13.8)
Constipation									
No	5.3 (3.8)	3.9 (3.1)	7.1 (3.0)	2.4 (2.0)	4.5 (2.1)	1.2 (1.4)	3.3 (2.0)	1.7 (1.5)	34.8 (14.0)
Yes	5.7 (3.9)	3.6 (2.7)	7.4 (3.0)	2.5 (1.8)	4.2 (2.3)	1.4 (1.5)	3.4 (2.2)	2.0 (1.6)	35.6 (14.0)
Reflux									
No	5.7 (3.7)	4.2 (3.0)	7.3 (3.0)	2.6 (1.9)	4.4 (2.2)	1.4 (1.4)	3.4 (2.0)	2.0 (1.6)	36.7 (14.1)
Yes	5.1 (4.0)	3.2 (2.9)	7.1 (2.9)	2.3 (2.0)	4.3 (2.0)	1.0 (1.4)	3.1 (2.0)	1.6 (1.5)	32.5 (13.2)
Insomnia									
Normal	4.8 (3.7)	3.7 (2.9)	7.1 (3.0)	2.3 (1.9)	4.2 (2.0)	0.9 (1.2)	3.0 (2.0)	1.8 (1.5)	32.7 (13.2)
Abnormal	7.0 (3.8)	4.1 (3.1)	7.5 (3.0)	2.8 (2.0)	5.0 (2.3)	2.0 (1.6)	4.0 (1.9)	1.8 (1.6)	40.7 (14.4)
Excessive daytime sleepiness									
Normal	4.9 (3.7)	3.6 (2.9)	6.8 (3.0)	2.2 (1.9)	4.2 (2.1)	1.1 (1.3)	3.0 (1.9)	1.7 (1.5)	32.5 (13.4)
Abnormal	7.1 (4.0)	4.6 (2.9)	8.5 (2.4)	3.1 (1.8)	5.2 (2.1)	1.8 (1.7)	4.4 (2.0)	2.1 (1.6)	43.3 (12.7)
Data source									
AussieRett	6.1 (3.7)	3.8 (2.9)	7.3 (3.0)	2.7 (1.9)	4.6 (2.2)	1.6 (1.4)	3.6 (2.1)	2.3 (1.5)	37.5 (13.8)
InterRett	5.0 (3.9)	3.8 (3.0)	7.2 (3.0)	2.2 (1.9)	4.2 (2.1)	1.0 (1.4)	3.1 (2.0)	1.5 (1.4)	33.3 (13.9)

* *n* = 362

SD, standard deviation

### Linear regression models

Table 3 presents the adjusted associations between the independent variables and the RSBQ total and selected subscale scores for the whole sample. Compared to individuals aged ≥ 25 years old, 2 to 12-year-old children had higher mean RSBQ Total (coefficient 4.21, 95% confidence interval [CI] 0.26,8.16); Night-time Behaviours (coefficient 0.43, 95% CI 0.02,0.83); and Fear/Anxiety (coefficient 0.65, 95% CI 0.05,1.25) scores. Compared to individuals with a C-terminal deletion, individuals with the p.Arg255* variant had higher mean Total (coefficient 5.50, 95% CI -0.48,11.49) and Night-time Behaviours (coefficient 0.68, 95% CI 0.06,1.29) scores, whereas the p.Arg294* variant had a higher mean Mood (coefficient 2.30, 95% CI 0.37,4.22) score. Compared to independent walking, the mean Total score was lower for individuals who were unable to walk (coefficient − 3.44, 95% CI -6.71,-0.17) and higher for individuals who walked with assistance (coefficient 3.69, 95% CI 0.31,7.08). Similarly, individuals with intermediate hand function abilities had a higher mean Total score (coefficient 3.78, 95% CI -0.21, 7.78). The mean Total and subscale scores were similar across categories for seizures, constipation, and reflux, but were higher in the presence of abnormal DIMS (Total: coefficient 3.69, 95% CI 0.31,7.08) and abnormal DOES (Total: coefficient 3.69, 95% CI 0.31,7.08) scores (Table 3).

**Table 3:** Adjusted* associations of covariates with selected RSBQ scores among individuals with Rett syndrome

	General Mood (0–16)	Breathing Problems (0–10)	Night-time Behaviours (0–6)	Fear/Anxiety (0–8)	Total (0–90)
n	365	365	365	365	362
	Adjusted coefficient (95% CI), *P*-value				
Age, in years					
2–12	0.65 (-0.47,1.78),0.25	0.80 (-0.10,1.69),0.08	0.43 (0.02,0.83),0.04	0.65 (0.05,1.25),0.03	4.21 (0.26,8.16),0.04
13–17	-0.49 (-1.60,0.62),0.38	0.24 (-0.64,1.13),0.59	-0.07 (-0.47,0.33),0.72	0.61 (0.01,1.20),0.04	1.34 (-2.58,5.26),0.50
18–24	-0.15 (-1.24,0.94),0.79	0.20 (-0.67,1.07),0.66	0.30 (-0.09,0.70),0.13	0.34 (-0.24,0.92),0.25	1.46 (-2.38,5.30),0.45
25+	Ref	Ref	Ref	Ref	Ref
Variant					
C-terminal deletion	Ref	Ref	Ref	Ref	Ref
Early truncation	0.57 (-1.28,2.41),0.55	0.40 (-1.08,1.87),0.60	0.33 (-0.34,1.00),0.33	-0.59 (-1.57,0.40),0.24	1.95 (-4.54,8.45),0.55
Large deletion	0.86 (-0.91,2.63),0.34	-0.04 (-1.46,1.37),0.95	0.30 (-0.34,0.94),0.36	-0.25 (-1.20,0.69),0.60	3.08 (-3.15,9.31),0.33
p.Arg106Trp	0.21 (-1.89,2.30),0.84	-0.70 (-2.38,0.98),0.41	0.09 (-0.67,0.85),0.82	-0.61 (-1.72,0.51),0.29	-0.06 (-7.44,7.32),0.99
p.Arg133Cys	1.20 (-0.57,2.98),0.18	-0.21 (-1.63,1.21),0.77	0.22 (-0.42,0.87),0.49	0.26 (-0.68,1.21),0.59	3.71 (-2.53,9.95),0.24
p.Thr158Met	1.06 (-0.62,2.73),0.21	0.05 (-1.29,1.39),0.95	0.31 (-0.30,0.91),0.32	0.21 (-0.68,1.10),0.64	3.36 (-2.54,9.25),0.26
p.Arg168*	0.39 (-1.25,2.03),0.64	-0.31 (-1.62,1.00),0.64	0.63 (0.03,1.22),0.04	0.31 (-0.56,1.19),0.48	3.26 (-2.52,9.03),0.27
p.Arg255*	1.19 (-0.51,2.89),0.17	0.60 (-0.76,1.96),0.39	0.68 (0.06,1.29),0.03	0.23 (-0.68,1.14),0.62	5.50 (-0.48,11.49),0.07
p.Arg270*	0.26 (-1.71,2.24),0.79	-0.63 (-2.21,0.95),0.43	0.32 (-0.40,1.03),0.38	-0.65 (-1.71,0.40),0.22	0.38 (-6.57,7.33),0.91
p.Arg294*	2.30 (0.37,4.22),0.02	-0.34 (-1.88,1.20),0.66	0.57 (-0.13,1.27),0.11	0.49 (-0.54,1.52),0.35	4.33 (-2.44,11.11),0.21
Arg306Cys	1.57 (-0.34,3.48),0.11	-0.59 (-2.12,0.94),0.45	0.69 (-0.00,1.38),0.05	0.69 (-0.33,1.70),0.19	2.77 (-4.04,9.58),0.42
Other	1.69 (0.01,3.37),0.05	0.51 (-0.84,1.85),0.46	0.30 (-0.31,0.91),0.34	-0.01 (-0.91,0.89),0.99	4.69 (-1.29,10.67),0.12
Unknown	0.04 (-2.32,2.40),0.97	-0.00 (-1.80,1.79),1.00	-0.40 (-1.26,0.46),0.36	-0.10 (-1.36,1.16),0.88	-0.03 (-8.20,8.14),0.99
Walking ability					
Unable	-0.95 (-1.88,-0.03),0.04	-0.32 (-1.06,0.42),0.40	-0.11 (-0.45,0.23),0.52	-0.01 (-0.51,0.48),0.96	-3.44 (-6.71,-0.17),0.04
Assisted	0.17 (-0.78,1.13),0.72	0.56 (-0.20,1.33),0.15	0.31 (-0.04,0.66),0.08	0.74 (0.23,1.25),<0.01	3.69 (0.31,7.08),0.03
Independent	Ref	Ref	Ref	Ref	Ref
Hand function					
Unable	-0.58 (-1.54,0.39),0.24	0.53 (-0.24,1.30),0.18	-0.05 (-0.40,0.30),0.77	0.07 (-0.45,0.58),0.80	2.79 (-0.61,6.20),0.11
Large objects	-0.50 (-1.62,0.63),0.39	0.78 (-0.12,1.69),0.09	-0.18 (-0.59,0.23),0.40	0.30 (-0.30,0.90),0.33	3.78 (-0.21,7.78),0.06
Small objects	Ref	Ref	Ref	Ref	Ref
Seizure frequency					
Never or controlled	Ref	Ref	Ref	Ref	Ref
Monthly or less	-0.66 (-1.58,0.26),0.16	0.68 (-0.05,1.42),0.07	-0.19 (-0.53,0.14),0.26	0.00 (-0.49,0.49),0.99	-0.24 (-3.48,3.01),0.89
Weekly	-0.35 (-1.55,0.85),0.57	1.43 (0.47,2.39),<0.01	0.26 (-0.17,0.70),0.23	-0.00 (-0.64,0.64),1.00	2.15 (-2.09,6.39),0.32
Daily	-1.05 (-2.33,0.23),0.11	0.42 (-0.61,1.44),0.42	0.07 (-0.39,0.54),0.76	-0.42 (-1.10,0.26),0.23	-2.37 (-6.92,2.19),0.31
Constipation					
No	Ref	Ref	Ref	Ref	Ref
Yes	-0.37 (-1.29,0.56),0.44	-0.44 (-1.18,0.30),0.24	-0.16 (-0.50,0.17),0.34	-0.26 (-0.76,0.23),0.29	-2.17 (-5.42,1.07),0.19
Reflux					
No	Ref	Ref	Ref	Ref	Ref
Yes	0.09 (-0.74,0.93),0.83	-0.86 (-1.53,-0.19),0.01	-0.01 (-0.32,0.29),0.93	0.04 (-0.41,0.49),0.86	-1.32 (-4.29,1.64),0.38
Insomnia					
Normal	Ref	Ref	Ref	Ref	Ref
Abnormal	1.82 (0.92,2.71),<0.01	0.13 (-0.59,0.85),0.72	1.03 (0.71,1.36),<0.01	0.59 (0.11,1.07),0.02	5.75 (2.58,8.92),<0.01
Excessive daytime sleepiness					
Normal	Ref	Ref	Ref	Ref	Ref
Abnormal	1.82 (0.84,2.79),<0.01	0.74 (-0.05,1.52),0.07	0.27 (-0.09,0.62),0.14	1.17 (0.65,1.69),<0.01	7.85 (4.39,11.31),<0.01
Data source					
AussieRett	Ref	Ref	Ref	Ref	Ref
InterRett	-0.78 (-1.73,0.18),0.11	0.12 (-0.65,0.89),0.76	-0.48 (-0.83,-0.13),0.01	-0.47 (-0.98,0.04),0.07	-3.89 (-7.28,-0.51),0.02

Ref, reference category; CI, confidence interval

* adjusted for all covariates

Table 4 presents the adjusted associations for the paediatric sub-sample. Compared to 13 to 17-year-old children, the mean RSBQ total score was higher for 2 to 12-year-old children (coefficient 3.65, 95% CI -0.61,7.90). Compared to C-terminal deletions, children with the p.Arg133Cys variant had the highest mean RSBQ Total score (coefficient 11.14, 95% CI 1.02, 21.27). Children with the p.Arg133Cys (coefficient 3.19, 95 CI 0.61, 5.78) and the p.Arg294* variants (coefficient 3.40, 95% CI 0.37, 6.43) had a higher mean General Mood score versus C-terminal deletions (Table 4). The mean RSBQ scores were broadly similar across the walking, hand function, seizures and reflux categories. Compared to children without constipation, children with constipation had higher mean Total, Breathing Problems, Night-time Behaviours and Fear/Anxiety scores. Abnormal DIMS scores were associated with higher mean Total (coefficient 4.83, 95% CI -0.15, 9.81) and General Mood and Night-time behaviours subscale scores. Abnormal DOES scores were associated with higher mean Total (coefficient 11.12, 95% CI 5.97, 16.27) and General Mood, Breathing Problems, Night-time Behaviours and Fear/Anxiety subscale scores (Table 4).

**Table 4:** Adjusted* associations of covariates with selected RSBQ scores in children with Rett syndrome

	General Mood (0–16)	Breathing Problems (0–10)	Night-time Behaviours (0–6)	Fear/Anxiety (0–8)	Total (0–90)
n	186	186	186	186	184
	Adjusted coefficient (95% CI), *P*-value				
Age, in years					
2–12	1.38 (0.29,2.46),0.01	0.60 (-0.32,1.51),0.20	0.57 (0.19,0.95),<0.01	0.23 (-0.36,0.83),0.44	3.65 (-0.61,7.90),0.09
13–17	Ref	Ref	Ref	Ref	Ref
Variant					
C-terminal deletion	Ref	Ref	Ref	Ref	Ref
Early truncation	1.41 (-1.03,3.85),0.26	0.80 (-1.25,2.86),0.44	0.23 (-0.63,1.09),0.60	-0.04 (-1.38,1.30),0.95	4.77 (-4.81,14.35),0.33
Large deletion	0.87 (-1.56,3.29),0.48	-0.04 (-2.07,2.00),0.97	0.23 (-0.62,1.09),0.59	0.67 (-0.66,1.99),0.32	4.48 (-5.01,13.97),0.35
p.Arg106Trp	2.94 (-0.48,6.37),0.09	0.27 (-2.61,3.15),0.85	0.66 (-0.55,1.86),0.28	-0.20 (-2.07,1.68),0.83	6.52 (-6.92,19.97),0.34
p.Arg133Cys	3.19 (0.61,5.78),0.02	0.62 (-1.55,2.80),0.57	0.61 (-0.30,1.52),0.18	0.47 (-0.94,1.89),0.51	11.14 (1.02,21.27),0.03
p.Thr158Met	1.69 (-0.69,4.07),0.16	0.73 (-1.28,2.73),0.47	0.53 (-0.31,1.36),0.22	0.36 (-0.94,1.66),0.59	7.10 (-2.23,16.43),0.13
p.Arg168*	-0.01 (-2.44,2.43),0.99	-0.35 (-2.40,1.70),0.74	0.40 (-0.46,1.26),0.36	0.37 (-0.96,1.71),0.58	2.04 (-7.51,11.59),0.67
p.Arg255*	0.85 (-1.66,3.36),0.51	1.24 (-0.87,3.36),0.25	0.14 (-0.74,1.03),0.75	0.37 (-1.00,1.75),0.59	5.24 (-4.60,15.08),0.29
p.Arg270*	0.19 (-2.41,2.78),0.89	-0.12 (-2.31,2.06),0.91	0.44 (-0.48,1.35),0.35	-0.77 (-2.20,0.65),0.29	2.58 (-7.61,12.77),0.62
p.Arg294*	3.40 (0.37,6.43),0.03	0.55 (-2.00,3.10),0.67	0.78 (-0.29,1.85),0.15	0.53 (-1.13,2.19),0.53	9.20 (-2.67,21.06),0.13
Arg306Cys	1.66 (-1.17,4.49),0.25	-1.16 (-3.54,1.22),0.34	0.64 (-0.35,1.64),0.20	0.93 (-0.62,2.48),0.24	1.88 (-9.21,12.97),0.74
Other	1.57 (-0.95,4.08),0.22	0.05 (-2.07,2.16),0.97	0.09 (-0.79,0.98),0.84	0.25 (-1.13,1.63),0.72	4.14 (-5.94,14.23),0.42
Unknown	0.57 (-2.57,3.71),0.72	-0.32 (-2.96,2.32),0.81	-0.38 (-1.48,0.73),0.50	0.02 (-1.70,1.74),0.98	2.46 (-9.84,14.76),0.69
Walking ability					
Unable	-1.00 (-2.29,0.28),0.13	-0.70 (-1.78,0.39),0.21	-0.34 (-0.79,0.11),0.14	-0.10 (-0.80,0.61),0.79	-3.16 (-8.21,1.89),0.22
Assisted	-0.67 (-2.10,0.76),0.36	0.13 (-1.08,1.33),0.84	0.06 (-0.44,0.57),0.81	0.77 (-0.01,1.55),0.05	0.71 (-4.95,6.37),0.81
Independent	Ref	Ref	Ref	Ref	Ref
Hand function					
Unable	-0.77 (-2.18,0.65),0.29	-0.25 (-1.44,0.94),0.68	-0.15 (-0.65,0.34),0.54	0.16 (-0.61,0.94),0.68	1.30 (-4.26,6.86),0.65
Large objects	-0.32 (-1.82,1.19),0.68	0.64 (-0.63,1.90),0.32	0.17 (-0.36,0.70),0.52	0.28 (-0.55,1.10),0.51	3.84 (-2.14,9.81),0.21
Small objects	Ref	Ref	Ref	Ref	Ref
Seizure frequency					
Never or controlled	Ref	Ref	Ref	Ref	Ref
Monthly or less	0.06 (-1.27,1.38),0.93	0.01 (-1.11,1.13),0.99	0.15 (-0.32,0.62),0.53	0.28 (-0.44,1.01),0.44	0.42 (-4.80,5.65),0.87
Weekly	0.02 (-1.66,1.69),0.98	0.96 (-0.45,2.37),0.18	0.42 (-0.17,1.01),0.16	0.36 (-0.55,1.28),0.44	3.10 (-3.47,9.67),0.35
Daily	-0.60 (-2.37,1.17),0.50	0.11 (-1.38,1.61),0.88	0.24 (-0.38,0.87),0.45	0.05 (-0.92,1.02),0.92	0.72 (-6.23,7.66),0.84
Constipation					
No	Ref	Ref	Ref	Ref	Ref
Yes	-0.63 (-1.91,0.66),0.34	-1.38 (-2.46,-0.30),0.01	-0.48 (-0.93,-0.02),0.04	-0.69 (-1.39,0.02),0.06	-5.96 (-10.99,-0.92),0.02
Reflux					
No	Ref	Ref	Ref	Ref	Ref
Yes	-0.18 (-1.36,1.00),0.77	-0.30 (-1.29,0.69),0.55	0.09 (-0.33,0.50),0.68	-0.20 (-0.85,0.45),0.54	-1.67 (-6.31,2.96),0.48
Insomnia					
Normal	Ref	Ref	Ref	Ref	Ref
Abnormal	1.50 (0.23,2.77),0.02	0.18 (-0.89,1.25),0.74	0.93 (0.48,1.38),<0.01	0.60 (-0.09,1.30),0.09	4.83 (-0.15,9.81),0.06
Excessive daytime sleepiness					
Normal	Ref	Ref	Ref	Ref	Ref
Abnormal	2.10 (0.78,3.41),<0.01	1.65 (0.54,2.75),<0.01	0.35 (-0.11,0.82),0.14	1.58 (0.86,2.30),<0.01	11.12 (5.97,16.27),<0.01
Data source					
AussieRett	Ref	Ref	Ref	Ref	Ref
InterRett	0.06 (-1.30,1.43),0.93	0.55 (-0.59,1.70),0.34	-0.54 (-1.02,-0.06),0.03	0.03 (-0.72,0.78),0.94	-1.46 (-6.81,3.90),0.59

Ref, reference category; CI, confidence interval

* adjusted for all covariates

Table 5 presents the adjusted associations for the adult sub-sample. The mean RSBQ Total scores were similar for adults younger and older than 25 years. Compared to the C-terminal deletion, adults with the p.Arg255* variant had the highest mean Total (coefficient 7.05, 95% CI -0.22,14.32) and Night-time Behaviours subscale (coefficient 1.31, 95% CI 0.41,2.21) scores, whereas the scores were similar for the other subscales. Adults unable to walk had a lower mean Total score (coefficient − 4.28, 95% CI -8.64,0.09), whereas adults who walked with assistance had a higher mean Total score (coefficient 7.09, 95% CI 2.92,11.27). Compared to no seizures, adults with daily seizures had a lower mean Total score (coefficient − 5.35, 95% CI -11.48,0.79). Abnormal DIMS scores were associated with higher mean Total, General Mood and Night-time Behaviours scores, whereas abnormal DOES scores had less influence on the RSBQ scores. Adults in the InterRett sample had lower mean Total, General Mood and Fear/Anxiety scores than adults in the AussieRett sample.

**Table 5:** Adjusted* associations of covariates with selected RSBQ scores in adults with Rett syndrome

	General Mood (0–16)	Breathing Problems (0–10)	Night-time Behaviours (0–6)	Fear/Anxiety (0–8)	Total (0–90)
n	179	179	179	179	178
	Adjusted coefficient (95% CI), *P*-value				
Age, in years					
18–24	-0.21 (-1.36,0.94),0.72	0.34 (-0.50,1.18),0.42	0.31 (-0.12,0.74),0.15	0.37 (-0.24,0.98),0.23	1.48 (-1.98,4.95),0.40
25+	Ref	Ref	Ref	Ref	Ref
Variant					
C-terminal deletion	Ref	Ref	Ref	Ref	Ref
Early truncation	-0.72 (-3.70,2.27),0.64	0.44 (-1.75,2.62),0.69	0.56 (-0.56,1.67),0.32	-1.35 (-2.93,0.23),0.09	-1.18 (-10.16,7.80),0.80
Large deletion	1.49 (-1.24,4.22),0.28	0.46 (-1.54,2.46),0.65	0.62 (-0.40,1.64),0.23	-1.32 (-2.77,0.12),0.07	3.43 (-4.77,11.64),0.41
p.Arg106Trp	-1.47 (-4.30,1.36),0.31	-0.55 (-2.62,1.52),0.60	-0.27 (-1.32,0.79),0.62	-0.59 (-2.09,0.91),0.44	-3.23 (-11.74,5.27),0.45
p.Arg133Cys	-0.49 (-2.99,2.01),0.70	-0.74 (-2.57,1.09),0.43	-0.04 (-0.97,0.90),0.94	0.02 (-1.31,1.34),0.98	-2.43 (-9.95,5.09),0.52
p.Thr158Met	0.06 (-2.42,2.54),0.96	0.00 (-1.81,1.82),1.00	0.03 (-0.90,0.95),0.95	0.23 (-1.09,1.54),0.74	-0.57 (-8.02,6.89),0.88
p.Arg168*	0.81 (-1.51,3.14),0.49	0.17 (-1.53,1.87),0.84	0.79 (-0.08,1.66),0.08	0.24 (-0.99,1.47),0.70	4.61 (-2.39,11.62),0.20
p.Arg255*	1.69 (-0.73,4.11),0.17	0.57 (-1.20,2.34),0.53	1.31 (0.41,2.21),<0.01	0.11 (-1.17,1.39),0.86	7.05 (-0.22,14.32),0.06
p.Arg270*	1.06 (-2.09,4.21),0.51	-1.36 (-3.67,0.95),0.25	0.29 (-0.89,1.47),0.63	-0.29 (-1.96,1.37),0.73	-2.13 (-11.61,7.35),0.66
p.Arg294*	1.56 (-1.08,4.20),0.25	-0.22 (-2.15,1.71),0.82	0.51 (-0.48,1.49),0.31	0.78 (-0.61,2.18),0.27	3.39 (-4.54,11.32),0.40
Arg306Cys	1.21 (-1.44,3.86),0.37	-0.40 (-2.34,1.54),0.68	0.62 (-0.37,1.61),0.22	0.29 (-1.11,1.69),0.68	2.21 (-5.96,10.38),0.59
Other	1.80 (-0.55,4.14),0.13	1.01 (-0.71,2.73),0.25	0.48 (-0.39,1.36),0.28	-0.18 (-1.42,1.06),0.78	4.86 (-2.20,11.92),0.18
Unknown	0.11 (-3.62,3.85),0.95	0.34 (-2.39,3.07),0.81	-0.28 (-1.68,1.11),0.69	0.17 (-1.81,2.14),0.87	-1.63 (-12.86,9.59),0.77
Walking ability					
Unable	-0.94 (-2.39,0.52),0.20	-0.29 (-1.36,0.77),0.59	0.05 (-0.49,0.59),0.86	-0.13 (-0.90,0.63),0.73	-4.28 (-8.64,0.09),0.05
Assisted	1.10 (-0.29,2.49),0.12	0.85 (-0.16,1.87),0.10	0.63 (0.11,1.15),0.02	0.73 (-0.00,1.47),0.05	7.09 (2.92,11.27),<0.01
Independent	Ref	Ref	Ref	Ref	Ref
Hand function					
Unable	-0.92 (-2.33,0.48),0.20	1.10 (0.07,2.13),0.04	-0.14 (-0.66,0.39),0.60	0.10 (-0.64,0.85),0.79	3.55 (-0.70,7.80),0.10
Large objects	-0.91 (-2.80,0.98),0.34	0.97 (-0.41,2.35),0.17	-0.63 (-1.33,0.08),0.08	0.40 (-0.60,1.40),0.43	4.92 (-0.79,10.62),0.09
Small objects	Ref	Ref	Ref	Ref	Ref
Seizure frequency					
Never or controlled	Ref	Ref	Ref	Ref	Ref
Monthly or less	-1.47 (-2.84,-0.10),0.04	1.26 (0.26,2.26),0.01	-0.47 (-0.99,0.04),0.07	-0.27 (-1.00,0.46),0.46	-1.10 (-5.22,3.03),0.60
Weekly	-0.49 (-2.33,1.34),0.60	1.72 (0.38,3.06),0.01	0.12 (-0.57,0.80),0.73	-0.52 (-1.49,0.45),0.29	1.39 (-4.13,6.90),0.62
Daily	-1.37 (-3.34,0.61),0.17	0.54 (-0.90,1.98),0.46	-0.15 (-0.88,0.59),0.69	-0.76 (-1.80,0.28),0.15	-5.35 (-11.48,0.79),0.09
Constipation					
No	Ref	Ref	Ref	Ref	Ref
Yes	-0.11 (-1.53,1.31),0.88	0.68 (-0.36,1.71),0.20	0.14 (-0.38,0.67),0.59	0.20 (-0.55,0.95),0.59	2.32 (-1.94,6.59),0.28
Reflux					
No	Ref	Ref	Ref	Ref	Ref
Yes	0.28 (-0.98,1.54),0.66	-1.40 (-2.32,-0.48),<0.01	-0.20 (-0.67,0.27),0.40	0.11 (-0.55,0.78),0.74	-1.79 (-5.61,2.03),0.35
Insomnia					
Normal	Ref	Ref	Ref	Ref	Ref
Abnormal	2.46 (1.08,3.85),<0.01	0.24 (-0.77,1.25),0.64	1.17 (0.65,1.69),<0.01	0.53 (-0.21,1.26),0.16	6.99 (2.82,11.16),<0.01
Excessive daytime sleepiness					
Normal	Ref	Ref	Ref	Ref	Ref
Abnormal	1.35 (-0.22,2.91),0.09	-0.38 (-1.53,0.76),0.51	0.16 (-0.43,0.74),0.60	0.71 (-0.11,1.54),0.09	2.78 (-1.96,7.52),0.25
Data source					
AussieRett	Ref	Ref	Ref	Ref	Ref
InterRett	-1.34 (-2.74,0.07),0.06	-0.27 (-1.30,0.76),0.61	-0.35 (-0.87,0.18),0.19	-0.97 (-1.71,-0.22),0.01	-5.29 (-9.52,-1.06),0.01

Ref, reference category; CI, confidence interval

* adjusted for all covariates

Supplementary Tables 3, 4 and 5 in Additional File 1 present the unadjusted results for the total and the paediatric and adult subgroups. Supplementary Table 6 in Additional File 1 presents the paediatric Total and subscale scores, and Supplementary Table 7 in Additional File 1 presents the adult Total and subscale scores, for the adjusted item sets from recent factor analyses [16] where patterns were broadly similar to the original factor structure described above.

## Discussion

This is the largest study to date to evaluate associations between genotype, phenotype and RSBQ scores in RTT. Generally, scores were higher for children, and for individuals with the p.Arg255* variant, intermediate walking and hand function abilities, and abnormal insomnia and excessive daytime sleepiness. Except for associations with sleep, many associations between age, genotype, functional abilities and RSBQ were inconsistent with literature evaluating clinical severity as an outcome in RTT. Our cross-sectional findings suggest different relationships between clinical severity, functional abilities and behaviours, as reflected by RSBQ scores.

We expected to find higher scores in adults than children because greater clinical severity has been observed in other cross-sectional studies [23, 24]. However, RSBQ Total, Night-time Behaviours and Fear/Anxiety scores were higher for children than adults, in line with the other large international sample study (323 children, 309 adults) [16]. This age-related pattern was not observed in a smaller UK study of children and adults (*n* = 91) [7]. Lower scores in adulthood could represent a survival bias or that behaviours such as symptoms associated with anxiety were calmer [25]. Not previously reported, our paediatric data showed higher Total, General Mood and Fear/Anxiety scores for children younger than 12 years compared with children 12 years of older. We know that heightened behavioural symptoms such as irritability occur around the regression period [26] and it could be that the following period of relative stability is characterised by a stronger behavioural phenotype than previously recognised. Longitudinal data in RTT shows that clinical severity is greater [27], gross motor skills are poorer [28] and scoliosis increases [29] with increasing age. In contrast, longitudinal data on sleep shows small improvements with age [30]. 

Individuals with the p.Arg255* variant had the highest Total and Night-Time Behaviour scores showing greater severity of behaviours that appear to accompany their greater clinical severity [4]. In contrast, other variant groups with high RSBQ Total scores were the p.Arg294* and p.Arg133Cys which are usually associated with milder clinical severity for developmental impairments and comorbidities [4]. The patterns between genotype and behaviours as represented in the RSBQ appear different to relationships with the previously investigated construct of clinical severity that represents developmental impairments and comorbidities [31]. We did observe that individuals with the p.Arg294* variant were vulnerable to high General Mood scores, observed also when the sample was restricted to children. This is consistent with previous analyses suggesting that this variant is associated with poorer mental health [8, 25]. 

When previously studying anxiety as an outcome using InterRett data [25], we observed that ADAMS scores were higher, indicating more behavioural difficulties, for individuals with intermediate levels of walking and hand function abilities, consistent with the current study. A similar lack of pattern was observed for relationships between frequency of seizures and RSBQ scores. These findings highlight the conceptual differences between behaviours and clinical severity, as also observed in the relationships between variants and RSBQ scores.

Insomnia and excessive daytime sleepiness had the strongest relationships with RSBQ Total scores and the General Mood, Breathing Problems, Night-Time Behaviours and Fear/Anxiety subscales. This is not surprising because of the importance for behaviours and mental health attributable to good quality sleep [32, 33]. Insomnia and excessive daytime sleepiness have negative impacts on mental health in individuals with RTT [25]. Downstream, poor sleep also has impacts on quality of life in RTT [34]. The Breathing Problems subscale in the RSBQ is unusual because it describes neurological rather that behavioural characteristics [14, 16], although hyperventilation may also be interpreted as indicating anxiety. In the current study, RSBQ scores for Breathing Problems were similar between variant groups in contrast to our previous report where parents rated greater impact of breathing abnormalities for individuals with the p.Arg294* variant [35]. For the whole sample, scores were similar for seizures and gastrointestinal problems. However, children with constipation had higher RSBQ scores compared to children without constipation. It could be that stepped regimens that families use to manage constipation [36] are explored during early childhood and become established as the child grows older, lessening any impacts on the non-verbal behaviours which are captured in the RSBQ.

Our data indicates that the RSBQ is an outcome measure of the behavioural rather than the clinical phenotype in RTT [14], as also acknowledged by Percy and colleagues in their recent publication evaluating the RSBQ as an outcome measure. As might be expected, the behavioural phenotype tends to reflect mental health rather than physical functioning. For example, it does not measure comorbidities of poor growth, gastrointestinal problems, scoliosis or epilepsy. In this dataset mental health symptomatology has tended to occur most in individuals with variants of milder severity such as p.Arg294*, p.Arg133Cys and p.Arg306Cys consistent with the patterns seen previously when assessing anxiety [25]. The phenotype of p.Arg255* is interesting because it is localised in the Nuclear Localising Signal in close proximity to p.Arg270*, a genotype associated with early mortality [37, 38] and thus more likely to feature less commonly in the adult population. In contrast the p.Arg255*, although associated with greater clinical severity [4], has been shown to provide some protection against mortality [39, 40]. 

it is important to recognise that the top concerns identified by caregivers of individuals with classic RTT were effective communication, seizures, walking/balance issues, lack of hand use, and constipation [41]. Of these, the only subscale clearly identified in the RSBQ is hand behaviours and there is no specific question as to whether the individual can walk independently. Communication other than eye gaze, seizures and constipation are not measured in the RSBQ. Nevertheless, the RSBQ measures a range of behaviours such as mood, anxiety and night-time behaviours that can be problematic and of great concern to parents. It is important to understand the strengths and limitations of the RSBQ when designing suites of outcome measures for clinical trials.

Although the total sample is not population-based, the ability to combine data from two different sources (total sample size 365) is a major strength of the study. We note however that the total data on independent mobility is consistent with the population data presented on trajectories in a 2016 study describing the natural history of scoliosis [29]. We have previously found that the proportion with a p.Arg255* variant is relatively high in InterRett [19] and hence in the combined sample. We selected covariates and RSBQ subscales for analysis which we felt would be meaningful to caregivers and which are reflected in top caregiver concerns [41], although we did not have granular measure of communication which is an important concern for caregivers [41]. We have previously acknowledged the survival effect associated with our InterRett data [39]. There is always going to be a survival effect associated with the adult phenotype but it is important to understand the applicability of the RSBQ to paediatric and adult populations.

## Conclusion

Our data contribute further to the evidence that the RSBQ measures the behavioural phenotype rather than the clinical severity in RTT as traditionally conceptualised in terms of developmental regression, functional abilities and comorbidities, although it does measure autonomic/breathing irregularities. Its strength is in the assessment of a complex set of behavioural problems including mood difficulties and anxiety as well as hand stereotypies. When designing clinical trials, the RSBQ needs to be complemented by other metrics [42] which adequately assess core functional difficulties and comorbidities [43] and, for additional perspectives, could also include global ratings of impressions of improvements [44]. Analyses of longitudinal RSBQ trajectories and their relationships with genotype and phenotype will also be an important next step to enrich understanding of the natural history of RTT and inform post-clinical trial surveillance.

## Electronic supplementary material

Below is the link to the electronic supplementary material.

Supplementary Material 1

## Data Availability

Deidentified individual participant data will be made available upon publication to researchers who provide a methodologically sound proposal for use and subject to ethical approval.

## Abbreviations

CI: Confidence intervals
DIMS: Disorders of Initiating and Maintaining Sleep
DOES: Disorders of Excessive Somnolence
MECP2: methyl CpG binding protein 2
RSBQ: Rett Syndrome Behaviour Questionnaire
RTT: Rett syndrome
SDSC: Sleep Disorder Scale for Children
