# Supplementary material for: Associations between genotype, phenotype and behaviours measured by the Rett syndrome behaviour questionnaire in Rett syndrome
Source: J Neurodev Disord. 2024 Oct 25;16:59. doi: 10.1186/s11689-024-09575-4 (PMC11515842; doi:10.1186/s11689-024-09575-4)
Supplement: Supplementary file 1 — Supplementary Material 1 [file 11689_2024_9575_MOESM1_ESM.docx]

**Additional file 1**

Tables presented as word documents - .docx

Names of tables:

1. Supplementary Table 1, Additional File 1. RSBQ scores (8-factor, Mount et al., 2002) by covariates in 186 children with Rett syndrome
2. Supplementary Table 2, Additional File 1. RSBQ scores (8-factor, Mount et al., 2002) by covariates in 179 adults with Rett syndrome
3. Supplementary Table 3, Additional File 1. Unadjusted associations of covariates with selected RSBQ scores in 365 individuals with Rett syndrome
4. Supplementary Table 4, Additional File 1. Unadjusted associations of covariates with selected RSBQ scores in 186 children with Rett syndrome
5. Supplementary Table 5, Additional File 1. Unadjusted associations of covariates with selected RSBQ scores in 179 adults with Rett syndrome
6. Supplementary Table 6, Additional File 1. RSBQ scores (6-factor, Oberman et al., 2023) by covariates in 186 children with Rett syndrome
7. Supplementary Table 7, Additional File 1. RSBQ scores (7-factor, Oberman et al., 2023) by covariates in 179 adults with Rett syndrome

**Additional File 1 Table 1. RSBQ scores (8-factor, Mount et al., 2002) by covariates in 186 children with Rett syndrome**

|  | **Subscale** | | | | | | | | **Total***  (0-90) |
| --- | --- | --- | --- | --- | --- | --- | --- | --- | --- |
| Factor (range) | **Factor 1**  General Mood  (0-16) | **Factor 2** Breathing Problems  (0-10) | **Factor 3**  Hand Behaviours  (0-12) | **Factor 4**  Face Movements  (0-8) | **Factor 5**  Body Rocking  (0-12) | **Factor 6**  Night-time Behaviours  (0-6) | **Factor 7**  Fear/Anxiety  (0-8) | **Factor 8***  Walking/  Standing  (0-4) |  |
|  | mean (SD) | | | | | | | | |
| Overall | 5.6 (3.8) | 4.1 (3.1) | 7.1 (3.0) | 2.8 (2.1) | 4.4 (2.2) | 1.3 (1.4) | 3.6 (2.0) | 1.8 (1.6) | 36.6 (14.9) |
| Age, in years |  |  |  |  |  |  |  |  |  |
| 2-12 | 6.4 (3.7) | 4.3 (2.8) | 6.8 (2.9) | 3.1 (2.1) | 4.5 (2.3) | 1.6 (1.5) | 3.7 (2.1) | 1.7 (1.6) | 38.5 (14.7) |
| 13-17 | 4.9 (3.8) | 3.9 (3.3) | 7.3 (3.0) | 2.4 (2.0) | 4.4 (2.0) | 0.9 (1.2) | 3.4 (1.9) | 1.8 (1.6) | 34.7 (15.0) |
| Variant |  |  |  |  |  |  |  |  |  |
| C-terminal | 4.3 (2.7) | 3.7 (3.1) | 6.3 (3.6) | 2.2 (1.8) | 4.3 (2.2) | 0.9 (1.3) | 3.3 (2.3) | 1.4 (1.3) | 31.0 (15.0) |
| Early truncation | 5.4 (3.8) | 4.4 (3.3) | 7.0 (3.3) | 2.5 (2.2) | 4.0 (2.3) | 1.1 (1.5) | 3.0 (2.3) | 2.3 (1.6) | 35.0 (17.3) |
| Large deletion | 4.9 (3.7) | 3.1 (3.6) | 5.6 (3.1) | 2.8 (2.0) | 4.5 (1.9) | 1.2 (1.5) | 3.5 (1.8) | 1.7 (1.7) | 33.3 (15.9) |
| p.Arg106Trp | 8.3 (5.1) | 4.7 (3.0) | 8.3 (3.6) | 1.9 (1.2) | 5.9 (3.5) | 1.9 (1.1) | 3.4 (1.5) | 2.1 (0.9) | 42.1 (16.6) |
| p.Arg133Cys | 7.2 (4.0) | 4.2 (2.6) | 7.7 (3.0) | 3.2 (2.8) | 5.0 (2.5) | 1.4 (1.3) | 3.4 (1.9) | 2.2 (1.6) | 40.9 (14.5) |
| p.Thr158Met | 5.9 (3.8) | 4.4 (3.1) | 7.5 (2.2) | 3.0 (1.9) | 4.4 (1.8) | 1.4 (1.3) | 3.5 (2.3) | 1.9 (1.7) | 38.2 (14.2) |
| p.Arg168* | 4.9 (2.8) | 3.9 (3.2) | 7.1 (3.0) | 2.8 (2.1) | 4.1 (2.3) | 1.7 (1.5) | 3.9 (2.2) | 1.3 (1.6) | 36.4 (15.0) |
| p.Arg255* | 5.0 (4.2) | 5.5 (3.5) | 7.3 (3.0) | 3.3 (2.3) | 4.2 (1.8) | 0.9 (1.0) | 4.0 (2.2) | 1.9 (1.7) | 38.6 (15.7) |
| p.Arg270* | 5.4 (4.1) | 4.1 (3.1) | 8.1 (2.3) | 3.1 (2.3) | 4.9 (2.1) | 1.7 (1.6) | 3.2 (1.9) | 1.8 (1.8) | 39.0 (16.6) |
| p.Arg294* | 7.8 (4.5) | 4.1 (2.9) | 7.2 (2.5) | 2.6 (2.3) | 5.0 (2.4) | 1.5 (1.7) | 3.6 (2.0) | 1.6 (1.5) | 39.0 (14.0) |
| Arg306Cys | 6.0 (2.9) | 2.7 (2.3) | 6.0 (3.2) | 2.1 (1.6) | 3.7 (1.8) | 1.6 (1.0) | 4.1 (2.1) | 1.1 (1.3) | 32.0 (13.9) |
| Other | 5.8 (3.9) | 3.9 (2.4) | 7.5 (3.0) | 2.4 (2.0) | 3.8 (2.4) | 0.9 (1.5) | 3.5 (1.8) | 1.7 (1.8) | 35.6 (12.6) |
| Unknown | 6.1 (5.1) | 4.1 (2.8) | 6.6 (1.1) | 3.6 (1.3) | 5.1 (1.7) | 1.1 (1.8) | 3.9 (1.9) | 2.3 (1.9) | 39.3 (12.8) |
| Walking ability |  |  |  |  |  |  |  |  |  |
| Unable | 5.4 (3.5) | 3.8 (3.0) | 7.2 (3.1) | 2.9 (2.0) | 4.4 (2.2) | 1.1 (1.3) | 3.4 (2.2) | 1.2 (1.5) | 35.4 (14.8) |
| Assisted | 5.1 (4.0) | 4.5 (3.1) | 7.1 (2.8) | 2.8 (2.3) | 4.3 (2.4) | 1.4 (1.5) | 4.1 (2.0) | 2.2 (1.5) | 37.7 (16.3) |
| Independent | 6.0 (3.8) | 4.0 (3.0) | 6.9 (2.9) | 2.6 (2.1) | 4.5 (2.0) | 1.3 (1.3) | 3.3 (1.9) | 2.0 (1.6) | 36.3 (14.0) |
| Hand function |  |  |  |  |  |  |  |  |  |
| Unable | 4.9 (3.2) | 4.0 (2.8) | 7.9 (2.8) | 2.8 (1.9) | 4.3 (2.1) | 1.0 (1.1) | 3.5 (2.1) | 1.8 (1.6) | 35.8 (12.1) |
| Large objects | 6.0 (4.4) | 5.0 (3.1) | 8.1 (2.3) | 3.0 (2.2) | 4.6 (2.3) | 1.5 (1.6) | 4.0 (2.0) | 1.8 (1.6) | 40.5 (16.4) |
| Small objects | 5.9 (3.8) | 3.5 (3.1) | 5.7 (3.0) | 2.5 (2.1) | 4.4 (2.1) | 1.3 (1.4) | 3.3 (2.0) | 1.7 (1.6) | 34.3 (15.4) |
| Seizure frequency |  |  |  |  |  |  |  |  |  |
| Never or controlled | 5.8 (3.7) | 3.9 (3.2) | 6.6 (3.0) | 2.8 (2.1) | 4.2 (2.2) | 1.1 (1.2) | 3.3 (2.0) | 1.6 (1.6) | 35.2 (14.4) |
| Monthly or less | 5.3 (3.7) | 3.9 (3.1) | 7.4 (3.1) | 2.5 (2.0) | 4.3 (2.1) | 1.1 (1.2) | 3.6 (2.0) | 1.7 (1.6) | 35.3 (14.5) |
| Weekly | 5.7 (4.1) | 4.9 (2.4) | 7.3 (2.7) | 3.2 (2.3) | 4.6 (2.5) | 1.6 (1.7) | 3.8 (2.0) | 2.0 (1.7) | 39.8 (16.4) |
| Daily | 5.8 (4.1) | 4.3 (3.1) | 7.3 (2.6) | 2.7 (1.9) | 4.8 (1.9) | 1.6 (1.6) | 4.0 (2.2) | 2.3 (1.5) | 39.6 (15.4) |
| Constipation |  |  |  |  |  |  |  |  |  |
| No | 5.6 (3.8) | 4.5 (3.2) | 7.1 (2.9) | 2.9 (2.1) | 4.5 (2.1) | 1.3 (1.3) | 3.7 (2.0) | 1.7 (1.6) | 37.2 (15.0) |
| Yes | 5.8 (3.8) | 3.3 (2.7) | 6.9 (3.1) | 2.5 (1.9) | 4.1 (2.3) | 1.4 (1.4) | 3.3 (2.0) | 2.0 (1.7) | 35.2 (15.0) |
| Reflux |  |  |  |  |  |  |  |  |  |
| No | 5.9 (3.7) | 4.2 (3.1) | 7.1 (3.0) | 2.9 (2.0) | 4.5 (2.2) | 1.4 (1.4) | 3.7 (2.0) | 2.1 (1.6) | 38.3 (15.4) |
| Yes | 5.1 (3.8) | 3.8 (3.0) | 6.9 (2.9) | 2.5 (2.1) | 4.3 (2.1) | 1.1 (1.3) | 3.2 (1.9) | 1.3 (1.4) | 33.7 (13.6) |
| Insomnia |  |  |  |  |  |  |  |  |  |
| Normal | 5.0 (3.7) | 4.0 (3.0) | 6.9 (2.9) | 2.6 (2.0) | 4.2 (2.2) | 0.9 (1.1) | 3.2 (2.0) | 1.8 (1.6) | 34.0 (14.3) |
| Abnormal | 6.9 (3.6) | 4.3 (3.1) | 7.5 (3.0) | 3.1 (2.1) | 4.9 (2.1) | 2.0 (1.6) | 4.2 (2.0) | 1.6 (1.6) | 41.9 (14.9) |
| Excessive daytime sleepiness |  |  |  |  |  |  |  |  |  |
| Normal | 5.0 (3.6) | 3.7 (3.0) | 6.5 (3.0) | 2.5 (2.1) | 4.1 (2.2) | 1.1 (1.2) | 3.1 (1.9) | 1.7 (1.5) | 33.0 (14.2) |
| Abnormal | 7.2 (3.9) | 5.1 (3.0) | 8.5 (2.5) | 3.4 (1.8) | 5.2 (1.9) | 1.8 (1.6) | 4.7 (1.9) | 2.0 (1.7) | 45.3 (13.1) |
| Data source |  |  |  |  |  |  |  |  |  |
| AussieRett | 6.0 (3.4) | 3.7 (2.9) | 6.9 (3.0) | 2.9 (1.9) | 4.6 (2.2) | 1.6 (1.4) | 3.6 (2.0) | 2.3 (1.6) | 37.8 (14.2) |
| InterRett | 5.4 (4.1) | 4.4 (3.1) | 7.2 (2.9) | 2.6 (2.2) | 4.2 (2.2) | 1.0 (1.3) | 3.6 (2.1) | 1.4 (1.5) | 35.7 (15.5) |

* n=184

SD, standard deviation

**Additional File 1 Table 2. RSBQ scores (8-factor, Mount et al., 2002) by covariates in 179 adults with Rett syndrome**

|  | **Subscale** | | | | | | | | **Total***  (0-90) |
| --- | --- | --- | --- | --- | --- | --- | --- | --- | --- |
| Factor (range) | **Factor 1**  General Mood  (0-16) | **Factor 2** Breathing Problems  (0-10) | **Factor 3**  Hand Behaviours  (0-12) | **Factor 4**  Face Movements  (0-8) | **Factor 5**  Body Rocking  (0-12) | **Factor 6**  Night-time Behaviours  (0-6) | **Factor 7**  Fear/Anxiety  (0-8) | **Factor 8***  Walking/  Standing  (0-4) |  |
|  | mean (SD) | | | | | | | | |
| Overall | 5.2 (3.9) | 3.5 (2.9) | 7.4 (3.0) | 2.1 (1.7) | 4.4 (2.1) | 1.2 (1.5) | 3.0 (2.0) | 1.8 (1.5) | 33.4 (12.8) |
| Age, in years |  |  |  |  |  |  |  |  |  |
| 18-24 | 5.4 (3.9) | 3.6 (2.8) | 7.5 (2.8) | 2.2 (1.7) | 4.5 (2.2) | 1.4 (1.6) | 3.3 (2.1) | 1.8 (1.5) | 34.5 (13.1) |
| 25+ | 5.1 (4.0) | 3.4 (2.9) | 7.2 (3.1) | 2.0 (1.7) | 4.2 (2.0) | 1.0 (1.4) | 2.8 (1.8) | 1.9 (1.5) | 32.4 (12.5) |
| Variant |  |  |  |  |  |  |  |  |  |
| C-terminal | 4.9 (3.7) | 3.8 (3.1) | 7.1 (3.0) | 2.2 (1.5) | 4.1 (2.1) | 1.0 (1.3) | 3.2 (2.3) | 1.6 (1.6) | 32.8 (11.5) |
| Early truncation | 4.0 (3.9) | 3.8 (3.4) | 8.9 (2.3) | 2.1 (1.6) | 4.2 (1.6) | 1.2 (1.7) | 1.9 (1.7) | 1.2 (1.3) | 32.2 (9.7) |
| Large deletion | 6.1 (3.6) | 4.4 (2.7) | 8.2 (2.7) | 2.5 (1.5) | 4.7 (1.6) | 1.4 (1.3) | 2.0 (1.4) | 2.5 (1.1) | 37.0 (9.1) |
| p.Arg106Trp | 4.0 (3.0) | 2.8 (2.9) | 7.4 (2.8) | 1.9 (1.1) | 4.7 (2.1) | 0.7 (1.3) | 2.6 (1.9) | 1.9 (1.5) | 30.9 (11.4) |
| p.Arg133Cys | 4.0 (3.9) | 2.0 (2.7) | 4.7 (3.0) | 1.6 (1.2) | 3.9 (1.8) | 0.7 (1.2) | 3.1 (2.0) | 1.7 (1.1) | 26.0 (13.1) |
| p.Thr158Met | 5.1 (4.1) | 3.5 (3.2) | 7.9 (3.0) | 1.5 (1.6) | 4.1 (2.1) | 1.0 (1.4) | 3.4 (2.2) | 2.4 (1.5) | 33.1 (12.8) |
| p.Arg168* | 4.7 (3.6) | 3.6 (3.3) | 8.6 (2.4) | 2.9 (2.2) | 4.0 (2.1) | 1.3 (1.5) | 3.2 (2.3) | 1.3 (1.6) | 35.1 (14.6) |
| p.Arg255* | 5.2 (5.0) | 3.4 (2.7) | 7.1 (3.5) | 2.1 (2.0) | 5.2 (2.5) | 1.7 (1.8) | 2.8 (1.9) | 1.7 (1.7) | 33.9 (17.4) |
| p.Arg270* | 5.2 (3.8) | 3.4 (2.4) | 8.2 (2.5) | 2.0 (2.1) | 3.4 (2.1) | 1.0 (1.4) | 3.0 (1.7) | 1.5 (1.9) | 33.4 (14.2) |
| p.Arg294* | 7.1 (3.3) | 2.6 (2.8) | 6.8 (3.1) | 1.2 (1.3) | 3.9 (2.1) | 1.7 (2.1) | 3.8 (1.6) | 1.6 (1.1) | 34.3 (10.4) |
| Arg306Cys | 6.8 (3.8) | 3.2 (2.3) | 7.9 (2.4) | 2.6 (1.8) | 5.4 (2.0) | 1.8 (2.0) | 4.0 (2.4) | 2.5 (1.3) | 37.1 (13.9) |
| Other | 6.0 (4.7) | 4.5 (2.3) | 7.4 (2.3) | 2.3 (1.6) | 4.4 (2.5) | 1.1 (1.1) | 2.8 (1.9) | 2.2 (1.5) | 35.9 (10.8) |
| Unknown | 5.0 (3.5) | 3.8 (3.7) | 5.3 (4.0) | 2.0 (1.9) | 4.7 (2.1) | 0.3 (0.5) | 2.8 (1.6) | 1.7 (1.4) | 31.0 (15.8) |
| Walking ability |  |  |  |  |  |  |  |  |  |
| Unable | 4.1 (3.4) | 3.0 (2.5) | 6.6 (2.7) | 1.9 (1.5) | 3.4 (1.7) | 1.0 (1.2) | 2.7 (1.9) | 0.7 (1.1) | 27.9 (9.2) |
| Assisted | 6.2 (4.2) | 4.2 (3.0) | 8.6 (2.6) | 2.3 (2.0) | 5.1 (2.1) | 1.5 (1.7) | 3.5 (1.9) | 2.6 (1.3) | 39.3 (12.9) |
| Independent | 5.3 (3.9) | 3.0 (2.9) | 6.7 (3.2) | 2.0 (1.6) | 4.5 (2.1) | 0.9 (1.3) | 2.7 (2.1) | 2.1 (1.3) | 32.0 (13.2) |
| Hand function |  |  |  |  |  |  |  |  |  |
| Unable | 4.7 (3.9) | 4.0 (2.9) | 8.1 (2.4) | 2.4 (1.8) | 4.3 (2.0) | 1.2 (1.6) | 2.8 (1.8) | 1.7 (1.7) | 33.8 (12.4) |
| Large objects | 5.4 (3.7) | 4.0 (3.1) | 9.4 (2.2) | 2.4 (1.6) | 4.9 (2.5) | 0.9 (1.0) | 3.3 (1.9) | 1.7 (1.4) | 37.7 (13.1) |
| Small objects | 5.8 (4.0) | 2.7 (2.6) | 5.8 (3.0) | 1.7 (1.5) | 4.3 (2.1) | 1.3 (1.6) | 3.2 (2.2) | 2.1 (1.2) | 31.3 (12.9) |
| Seizure frequency |  |  |  |  |  |  |  |  |  |
| Never or controlled | 5.5 (4.2) | 2.5 (2.6) | 6.9 (3.2) | 1.9 (1.7) | 4.5 (2.1) | 1.2 (1.5) | 3.1 (2.2) | 1.7 (1.4) | 32.2 (13.8) |
| Monthly or less | 4.5 (3.8) | 4.3 (2.7) | 7.8 (2.6) | 2.4 (1.7) | 4.2 (2.0) | 0.9 (1.1) | 2.9 (1.8) | 2.1 (1.5) | 33.8 (10.7) |
| Weekly | 5.3 (2.8) | 4.7 (3.1) | 8.1 (2.9) | 2.3 (1.7) | 4.4 (2.5) | 1.4 (1.6) | 3.0 (2.1) | 1.9 (1.7) | 36.7 (14.2) |
| Daily | 5.3 (4.2) | 4.1 (3.1) | 7.7 (2.6) | 2.1 (1.4) | 4.0 (1.7) | 1.5 (2.0) | 2.7 (1.7) | 1.7 (1.4) | 32.8 (10.0) |
| Constipation |  |  |  |  |  |  |  |  |  |
| No | 5.1 (3.9) | 3.3 (2.9) | 7.2 (3.0) | 1.9 (1.6) | 4.4 (2.0) | 1.1 (1.5) | 2.9 (1.9) | 1.8 (1.5) | 32.4 (12.7) |
| Yes | 5.6 (4.0) | 3.9 (2.8) | 7.9 (2.8) | 2.6 (1.7) | 4.3 (2.3) | 1.4 (1.5) | 3.5 (2.3) | 1.9 (1.5) | 36.0 (12.8) |
| Reflux |  |  |  |  |  |  |  |  |  |
| No | 5.4 (3.7) | 4.1 (2.9) | 7.5 (3.0) | 2.2 (1.6) | 4.4 (2.2) | 1.4 (1.5) | 3.1 (2.0) | 1.8 (1.5) | 35.0 (12.6) |
| Yes | 5.0 (4.3) | 2.5 (2.6) | 7.3 (2.9) | 1.9 (1.8) | 4.3 (2.0) | 1.0 (1.5) | 2.9 (2.1) | 1.9 (1.4) | 31.2 (12.8) |
| Insomnia |  |  |  |  |  |  |  |  |  |
| Normal | 4.6 (3.7) | 3.4 (2.8) | 7.3 (3.0) | 2.0 (1.6) | 4.1 (1.9) | 0.9 (1.3) | 2.9 (2.0) | 1.8 (1.5) | 31.6 (12.0) |
| Abnormal | 7.1 (4.0) | 3.8 (3.0) | 7.5 (3.0) | 2.4 (1.8) | 5.1 (2.6) | 2.0 (1.8) | 3.5 (1.8) | 2.0 (1.5) | 39.1 (13.6) |
| Excessive daytime sleepiness |  |  |  |  |  |  |  |  |  |
| Normal | 4.8 (3.8) | 3.4 (2.9) | 7.1 (3.0) | 2.0 (1.7) | 4.2 (2.0) | 1.1 (1.4) | 2.8 (1.9) | 1.8 (1.5) | 32.0 (12.7) |
| Abnormal | 7.1 (4.2) | 3.7 (2.6) | 8.5 (2.5) | 2.6 (1.6) | 5.0 (2.4) | 1.7 (1.8) | 4.0 (2.1) | 2.1 (1.5) | 40.0 (11.5) |
| Data source |  |  |  |  |  |  |  |  |  |
| AussieRett | 6.2 (4.0) | 3.9 (2.9) | 7.7 (2.9) | 2.5 (1.8) | 4.6 (2.2) | 1.5 (1.5) | 3.6 (2.2) | 2.3 (1.5) | 37.2 (13.4) |
| InterRett | 4.6 (3.8) | 3.2 (2.8) | 7.2 (3.0) | 1.9 (1.6) | 4.2 (2.0) | 1.0 (1.4) | 2.6 (1.8) | 1.5 (1.4) | 31.1 (11.9) |

* n=178

SD, standard deviation

**Additional File 1 Table 3. Unadjusted associations of covariates with selected RSBQ scores in 365 individuals with Rett syndrome**

|  | **General Mood**  (0-16) | **Breathing Problems**  (0-10) | **Night-time Behaviours**  (0-6) | **Fear/Anxiety**  (0-8) | **Total**  (0-90) |
| --- | --- | --- | --- | --- | --- |
| n | 365 | 365 | 365 | 365 | 362 |
|  | Adjusted β (95% CI), *P*-value | | | | |
| Age, in years |  |  |  |  |  |
| 2-12 | 1.28 (0.16,2.41),0.02 | 0.89 (0.02,1.75),0.05 | 0.67 (0.25,1.08),<0.01 | 0.91 (0.33,1.50),<0.01 | 6.14 (2.07,10.21),<0.01 |
| 13-17 | -0.24 (-1.35,0.87),0.67 | 0.56 (-0.29,1.42),0.20 | -0.03 (-0.44,0.38),0.88 | 0.68 (0.09,1.26),0.02 | 2.34 (-1.69,6.36),0.25 |
| 18-24 | 0.23 (-0.90,1.35),0.69 | 0.17 (-0.70,1.04),0.70 | 0.43 (0.02,0.85),0.04 | 0.51 (-0.08,1.11),0.09 | 2.14 (-1.95,6.24),0.30 |
| 25+ | Ref | Ref | Ref | Ref | Ref |
| Variant |  |  |  |  |  |
| C-terminal | Ref | Ref | Ref | Ref | Ref |
| Early truncation | 0.27 (-1.66,2.20),0.78 | 0.40 (-1.09,1.89),0.59 | 0.14 (-0.57,0.86),0.70 | -0.62 (-1.63,0.40),0.23 | 2.08 (-4.97,9.14),0.56 |
| Large deletion | 0.74 (-1.14,2.63),0.44 | -0.16 (-1.62,1.29),0.83 | 0.28 (-0.42,0.97),0.44 | -0.30 (-1.30,0.69),0.55 | 2.78 (-4.13,9.68),0.43 |
| p.Arg106Trp | 1.05 (-1.12,3.22),0.34 | -0.21 (-1.89,1.46),0.80 | 0.19 (-0.61,1.00),0.64 | -0.29 (-1.43,0.86),0.62 | 3.30 (-4.64,11.24),0.41 |
| p.Arg133Cys | 0.94 (-0.93,2.80),0.32 | -0.70 (-2.14,0.74),0.34 | 0.06 (-0.63,0.75),0.86 | 0.01 (-0.97,0.99),0.98 | 1.22 (-5.62,8.05),0.73 |
| p.Thr158Met | 0.94 (-0.84,2.73),0.30 | 0.23 (-1.15,1.61),0.74 | 0.22 (-0.45,0.88),0.52 | 0.21 (-0.73,1.15),0.66 | 3.97 (-2.57,10.50),0.23 |
| p.Arg168* | 0.18 (-1.53,1.90),0.83 | -0.04 (-1.37,1.28),0.95 | 0.53 (-0.11,1.16),0.10 | 0.32 (-0.58,1.22),0.49 | 3.75 (-2.52,10.02),0.24 |
| p.Arg255* | 0.52 (-1.22,2.25),0.56 | 0.70 (-0.64,2.05),0.30 | 0.33 (-0.31,0.97),0.32 | 0.22 (-0.70,1.13),0.64 | 4.45 (-1.90,10.80),0.17 |
| p.Arg270* | 0.75 (-1.28,2.78),0.47 | 0.09 (-1.47,1.66),0.91 | 0.48 (-0.27,1.23),0.21 | -0.09 (-1.16,0.98),0.86 | 4.88 (-2.66,12.42),0.20 |
| p.Arg294* | 2.72 (0.66,4.78),0.01 | -0.58 (-2.17,1.01),0.48 | 0.64 (-0.12,1.41),0.10 | 0.48 (-0.60,1.57),0.38 | 4.14 (-3.40,11.69),0.28 |
| Arg306Cys | 1.79 (-0.24,3.82),0.08 | -0.81 (-2.38,0.75),0.31 | 0.71 (-0.04,1.46),0.07 | 0.81 (-0.26,1.88),0.14 | 2.69 (-4.85,10.23),0.48 |
| Other | 1.27 (-0.50,3.04),0.16 | 0.49 (-0.88,1.86),0.48 | 0.03 (-0.63,0.68),0.94 | -0.09 (-1.02,0.85),0.85 | 3.79 (-2.75,10.33),0.25 |
| Unknown | 1.00 (-1.44,3.44),0.42 | 0.23 (-1.65,2.11),0.81 | -0.21 (-1.11,0.70),0.66 | 0.15 (-1.13,1.44),0.81 | 3.49 (-5.44,12.41),0.44 |
| Walking ability |  |  |  |  |  |
| Unable | -0.91 (-1.86,0.05),0.06 | -0.11 (-0.84,0.63),0.78 | -0.04 (-0.40,0.31),0.82 | 0.08 (-0.43,0.59),0.75 | -2.40 (-5.83,1.04),0.17 |
| Assisted | 0.06 (-0.91,1.04),0.90 | 0.79 (0.04,1.54),0.04 | 0.35 (-0.02,0.71),0.06 | 0.76 (0.25,1.27),<0.01 | 4.32 (0.81,7.83),0.02 |
| Independent | Ref | Ref | Ref | Ref | Ref |
| Hand function |  |  |  |  |  |
| Unable | -1.13 (-2.03,-0.24),0.01 | 0.84 (0.16,1.52),0.02 | -0.20 (-0.53,0.14),0.25 | -0.19 (-0.66,0.29),0.44 | 1.80 (-1.44,5.03),0.28 |
| Large objects | -0.09 (-1.13,0.96),0.87 | 1.55 (0.76,2.34),<0.01 | 0.01 (-0.37,0.40),0.94 | 0.51 (-0.04,1.06),0.07 | 6.69 (2.91,10.48),<0.01 |
| Small objects | Ref | Ref | Ref | Ref | Ref |
| Seizure frequency |  |  |  |  |  |
| Never or controlled | Ref | Ref | Ref | Ref | Ref |
| Monthly or less | -0.68 (-1.62,0.27),0.16 | 0.97 (0.25,1.69),0.01 | -0.14 (-0.49,0.21),0.42 | 0.10 (-0.40,0.60),0.69 | 1.21 (-2.22,4.63),0.49 |
| Weekly | -0.17 (-1.36,1.03),0.78 | 1.68 (0.78,2.58),<0.01 | 0.36 (-0.08,0.80),0.11 | 0.27 (-0.36,0.90),0.41 | 4.92 (0.58,9.26),0.03 |
| Daily | -0.10 (-1.39,1.19),0.88 | 1.10 (0.13,2.07),0.03 | 0.40 (-0.07,0.88),0.10 | 0.28 (-0.40,0.96),0.42 | 3.33 (-1.36,8.02),0.16 |
| Constipation |  |  |  |  |  |
| No | Ref | Ref | Ref | Ref | Ref |
| Yes | 0.42 (-0.45,1.29),0.34 | -0.31 (-0.98,0.36),0.37 | 0.21 (-0.11,0.53),0.20 | 0.13 (-0.33,0.58),0.59 | 0.83 (-2.34,4.00),0.61 |
| Reflux |  |  |  |  |  |
| No | Ref | Ref | Ref | Ref | Ref |
| Yes | -0.61 (-1.42,0.19),0.13 | -0.96 (-1.58,-0.34),<0.01 | -0.36 (-0.66,-0.07),0.02 | -0.34 (-0.77,0.08),0.11 | -4.20 (-7.11,-1.29),<0.01 |
| Insomnia |  |  |  |  |  |
| Normal | Ref | Ref | Ref | Ref | Ref |
| Abnormal | 2.19 (1.33,3.04),<0.01 | 0.46 (-0.22,1.14),0.18 | 1.10 (0.80,1.41),<0.01 | 0.92 (0.46,1.37),<0.01 | 8.03 (4.93,11.12),<0.01 |
| Excessive daytime sleepiness |  |  |  |  |  |
| Normal | Ref | Ref | Ref | Ref | Ref |
| Abnormal | 2.21 (1.30,3.12),<0.01 | 1.01 (0.29,1.72),0.01 | 0.68 (0.34,1.02),<0.01 | 1.45 (0.98,1.92),<0.01 | 10.83 (7.61,14.06),<0.01 |
| Data source |  |  |  |  |  |
| AussieRett | Ref | Ref | Ref | Ref | Ref |
| InterRett | -1.12 (-1.93,-0.32),0.01 | -0.00 (-0.63,0.62),0.99 | -0.59 (-0.88,-0.29),<0.01 | -0.48 (-0.90,-0.06),0.03 | -4.15 (-7.07,-1.22),0.01 |

Ref, reference category; CI, confidence interval

**Additional File 1 Table 4. Unadjusted associations of covariates with selected RSBQ scores in 186 children with Rett syndrome**

|  | **General Mood**  (0-16) | **Breathing Problems**  (0-10) | **Night-time Behaviours**  (0-6) | **Fear/Anxiety**  (0-8) | **Total**  (0-90) |
| --- | --- | --- | --- | --- | --- |
| n | 186 | 186 | 186 | 186 | 184 |
|  | Adjusted β (95% CI), *P*-value | | | | |
| Age, in years |  |  |  |  |  |
| 2-12 | 1.52 (0.44,2.60),0.01 | 0.32 (-0.56,1.21),0.47 | 0.70 (0.31,1.08),<0.01 | 0.24 (-0.35,0.83),0.42 | 3.80 (-0.52,8.13),0.08 |
| 13-17 | Ref | Ref | Ref | Ref | Ref |
| Variant |  |  |  |  |  |
| C-terminal | Ref | Ref | Ref | Ref | Ref |
| Early truncation | 1.08 (-1.45,3.60),0.40 | 0.66 (-1.40,2.72),0.53 | 0.11 (-0.81,1.04),0.81 | -0.28 (-1.66,1.10),0.69 | 4.03 (-6.06,14.12),0.43 |
| Large deletion | 0.60 (-1.92,3.13),0.64 | -0.60 (-2.66,1.45),0.56 | 0.23 (-0.69,1.16),0.62 | 0.25 (-1.13,1.63),0.72 | 2.29 (-7.79,12.38),0.65 |
| p.Arg106Trp | 4.01 (0.68,7.33),0.02 | 0.99 (-1.72,3.70),0.47 | 0.91 (-0.31,2.13),0.14 | 0.15 (-1.67,1.97),0.87 | 11.14 (-2.14,24.43),0.10 |
| p.Arg133Cys | 2.94 (0.28,5.60),0.03 | 0.49 (-1.68,2.66),0.65 | 0.48 (-0.49,1.46),0.33 | 0.15 (-1.30,1.61),0.84 | 9.89 (-0.73,20.52),0.07 |
| p.Thr158Met | 1.62 (-0.84,4.07),0.20 | 0.65 (-1.35,2.65),0.52 | 0.42 (-0.48,1.32),0.35 | 0.20 (-1.15,1.54),0.77 | 7.18 (-2.63,16.99),0.15 |
| p.Arg168* | 0.62 (-1.84,3.07),0.62 | 0.17 (-1.83,2.17),0.87 | 0.74 (-0.16,1.64),0.11 | 0.67 (-0.67,2.01),0.33 | 5.45 (-4.36,15.26),0.27 |
| p.Arg255* | 0.77 (-1.65,3.20),0.53 | 1.73 (-0.25,3.70),0.09 | -0.02 (-0.91,0.87),0.97 | 0.77 (-0.55,2.10),0.25 | 7.65 (-2.04,17.34),0.12 |
| p.Arg270* | 1.15 (-1.51,3.81),0.39 | 0.42 (-1.75,2.59),0.70 | 0.77 (-0.21,1.75),0.12 | -0.06 (-1.52,1.39),0.93 | 8.00 (-2.85,18.85),0.15 |
| p.Arg294* | 3.47 (0.30,6.65),0.03 | 0.40 (-2.18,2.99),0.76 | 0.56 (-0.61,1.72),0.35 | 0.35 (-1.39,2.08),0.69 | 8.00 (-4.67,20.67),0.21 |
| Arg306Cys | 1.72 (-1.22,4.67),0.25 | -1.02 (-3.42,1.38),0.40 | 0.66 (-0.42,1.74),0.23 | 0.82 (-0.79,2.43),0.31 | 1.00 (-10.76,12.76),0.87 |
| Other | 1.47 (-1.09,4.04),0.26 | 0.22 (-1.87,2.30),0.84 | -0.07 (-1.01,0.87),0.88 | 0.22 (-1.18,1.62),0.75 | 4.63 (-5.79,15.06),0.38 |
| Unknown | 1.87 (-1.46,5.19),0.27 | 0.42 (-2.29,3.13),0.76 | 0.20 (-1.02,1.42),0.75 | 0.58 (-1.24,2.40),0.53 | 8.29 (-5.00,21.57),0.22 |
| Walking ability |  |  |  |  |  |
| Unable | -0.66 (-1.93,0.62),0.31 | -0.19 (-1.23,0.84),0.71 | -0.16 (-0.63,0.31),0.51 | 0.18 (-0.51,0.87),0.61 | -0.90 (-5.98,4.18),0.73 |
| Assisted | -0.90 (-2.29,0.49),0.20 | 0.55 (-0.57,1.67),0.33 | 0.12 (-0.39,0.64),0.63 | 0.85 (0.10,1.60),0.03 | 1.45 (-4.11,7.01),0.61 |
| Independent | Ref | Ref | Ref | Ref | Ref |
| Hand function |  |  |  |  |  |
| Unable | -1.07 (-2.39,0.24),0.11 | 0.43 (-0.61,1.48),0.41 | -0.32 (-0.80,0.15),0.18 | 0.16 (-0.54,0.86),0.65 | 1.50 (-3.65,6.66),0.57 |
| Large objects | 0.05 (-1.28,1.38),0.94 | 1.48 (0.42,2.53),0.01 | 0.22 (-0.26,0.70),0.37 | 0.72 (0.01,1.42),0.05 | 6.15 (0.92,11.39),0.02 |
| Small objects | Ref | Ref | Ref | Ref | Ref |
| Seizure frequency |  |  |  |  |  |
| Never or controlled | Ref | Ref | Ref | Ref | Ref |
| Monthly or less | -0.50 (-1.83,0.82),0.45 | -0.01 (-1.07,1.05),0.98 | 0.03 (-0.45,0.50),0.91 | 0.29 (-0.41,0.99),0.42 | 0.10 (-5.09,5.28),0.97 |
| Weekly | -0.18 (-1.84,1.48),0.83 | 0.98 (-0.35,2.31),0.15 | 0.51 (-0.08,1.11),0.09 | 0.53 (-0.35,1.41),0.24 | 4.62 (-1.96,11.19),0.17 |
| Daily | -0.04 (-1.79,1.70),0.96 | 0.42 (-0.97,1.82),0.55 | 0.49 (-0.13,1.12),0.12 | 0.73 (-0.20,1.65),0.12 | 4.38 (-2.45,11.21),0.21 |
| Constipation |  |  |  |  |  |
| No | Ref | Ref | Ref | Ref | Ref |
| Yes | 0.27 (-0.91,1.45),0.66 | -1.18 (-2.12,-0.25),0.01 | 0.11 (-0.31,0.54),0.60 | -0.36 (-0.98,0.27),0.27 | -1.96 (-6.63,2.72),0.41 |
| Reflux |  |  |  |  |  |
| No | Ref | Ref | Ref | Ref | Ref |
| Yes | -0.82 (-1.92,0.29),0.15 | -0.39 (-1.29,0.51),0.39 | -0.34 (-0.74,0.06),0.10 | -0.48 (-1.07,0.10),0.11 | -4.65 (-9.00,-0.29),0.04 |
| Insomnia |  |  |  |  |  |
| Normal | Ref | Ref | Ref | Ref | Ref |
| Abnormal | 1.88 (0.74,3.03),<0.01 | 0.37 (-0.58,1.32),0.44 | 1.13 (0.74,1.53),<0.01 | 1.02 (0.41,1.63),<0.01 | 7.98 (3.48,12.48),<0.01 |
| Excessive daytime sleepiness |  |  |  |  |  |
| Normal | Ref | Ref | Ref | Ref | Ref |
| Abnormal | 2.14 (0.96,3.32),<0.01 | 1.42 (0.46,2.38),<0.01 | 0.67 (0.24,1.11),<0.01 | 1.54 (0.93,2.15),<0.01 | 12.24 (7.78,16.71),<0.01 |
| Data source |  |  |  |  |  |
| AussieRett | Ref | Ref | Ref | Ref | Ref |
| InterRett | -0.69 (-1.80,0.42),0.22 | 0.72 (-0.17,1.61),0.11 | -0.62 (-1.01,-0.22),<0.01 | 0.01 (-0.58,0.61),0.96 | -2.12 (-6.53,2.28),0.34 |

Ref, reference category; CI, confidence interval

**Additional File 1 Table 5. Unadjusted associations of covariates with selected RSBQ scores in 179 adults with Rett syndrome**

|  | **General Mood**  (0-16) | **Breathing Problems**  (0-10) | **Night-time Behaviours**  (0-6) | **Fear/Anxiety**  (0-8) | **Total**  (0-90) |
| --- | --- | --- | --- | --- | --- |
| n | 179 | 179 | 179 | 179 | 178 |
|  | Adjusted β (95% CI), *P*-value | | | | |
| Age, in years |  |  |  |  |  |
| 18-24 | 0.23 (-0.94,1.39),0.70 | 0.17 (-0.67,1.02),0.69 | 0.43 (-0.00,0.87),0.05 | 0.51 (-0.08,1.10),0.09 | 2.14 (-1.64,5.93),0.27 |
| 25+ | Ref | Ref | Ref | Ref | Ref |
| Variant |  |  |  |  |  |
| C-terminal | Ref | Ref | Ref | Ref | Ref |
| Early truncation | -0.90 (-4.02,2.21),0.57 | -0.03 (-2.30,2.23),0.98 | 0.22 (-0.95,1.39),0.71 | -1.30 (-2.88,0.28),0.11 | -0.59 (-10.77,9.59),0.91 |
| Large deletion | 1.19 (-1.72,4.10),0.42 | 0.55 (-1.56,2.67),0.61 | 0.36 (-0.73,1.46),0.51 | -1.19 (-2.67,0.29),0.11 | 4.19 (-5.32,13.70),0.39 |
| p.Arg106Trp | -0.90 (-3.81,2.00),0.54 | -0.99 (-3.11,1.12),0.36 | -0.27 (-1.37,0.82),0.62 | -0.55 (-2.03,0.92),0.46 | -1.90 (-11.41,7.61),0.69 |
| p.Arg133Cys | -0.90 (-3.55,1.74),0.50 | -1.81 (-3.73,0.11),0.06 | -0.33 (-1.33,0.66),0.51 | -0.12 (-1.47,1.22),0.86 | -6.81 (-15.45,1.83),0.12 |
| p.Thr158Met | 0.23 (-2.41,2.87),0.86 | -0.28 (-2.20,1.65),0.78 | -0.03 (-1.03,0.96),0.95 | 0.21 (-1.13,1.55),0.76 | 0.29 (-8.35,8.93),0.95 |
| p.Arg168* | -0.19 (-2.60,2.22),0.88 | -0.24 (-1.99,1.52),0.79 | 0.33 (-0.57,1.24),0.47 | -0.00 (-1.23,1.23),1.00 | 2.26 (-5.62,10.15),0.57 |
| p.Arg255* | 0.32 (-2.19,2.83),0.80 | -0.42 (-2.25,1.41),0.65 | 0.72 (-0.22,1.67),0.13 | -0.41 (-1.69,0.86),0.52 | 1.13 (-7.07,9.34),0.79 |
| p.Arg270* | 0.35 (-2.90,3.59),0.83 | -0.43 (-2.80,1.93),0.72 | -0.00 (-1.22,1.22),1.00 | -0.19 (-1.84,1.46),0.82 | 0.57 (-10.05,11.18),0.92 |
| p.Arg294* | 2.17 (-0.59,4.93),0.12 | -1.19 (-3.20,0.81),0.24 | 0.69 (-0.34,1.73),0.19 | 0.58 (-0.82,1.98),0.42 | 1.54 (-7.48,10.55),0.74 |
| Arg306Cys | 1.85 (-0.98,4.67),0.20 | -0.64 (-2.70,1.41),0.54 | 0.75 (-0.31,1.81),0.17 | 0.81 (-0.63,2.25),0.27 | 4.28 (-5.23,13.79),0.38 |
| Other | 1.10 (-1.38,3.57),0.38 | 0.72 (-1.08,2.52),0.43 | 0.11 (-0.82,1.04),0.82 | -0.35 (-1.61,0.91),0.58 | 3.06 (-5.03,11.15),0.46 |
| Unknown | 0.10 (-3.52,3.71),0.96 | 0.02 (-2.61,2.66),0.99 | -0.67 (-2.03,0.69),0.33 | -0.36 (-2.19,1.48),0.70 | -1.81 (-13.64,10.02),0.76 |
| Walking ability |  |  |  |  |  |
| Unable | -1.19 (-2.62,0.25),0.11 | 0.02 (-1.04,1.07),0.97 | 0.10 (-0.45,0.65),0.72 | -0.03 (-0.76,0.71),0.95 | -4.07 (-8.52,0.38),0.07 |
| Assisted | 0.94 (-0.45,2.32),0.18 | 1.18 (0.16,2.19),0.02 | 0.59 (0.06,1.11),0.03 | 0.81 (0.10,1.52),0.03 | 7.35 (3.05,11.65),<0.01 |
| Independent | Ref | Ref | Ref | Ref | Ref |
| Hand function |  |  |  |  |  |
| Unable | -1.15 (-2.41,0.11),0.07 | 1.27 (0.37,2.17),0.01 | -0.10 (-0.58,0.37),0.67 | -0.41 (-1.05,0.24),0.21 | 2.56 (-1.53,6.65),0.22 |
| Large objects | -0.42 (-2.17,1.33),0.63 | 1.31 (0.06,2.56),0.04 | -0.41 (-1.07,0.26),0.23 | 0.06 (-0.84,0.96),0.89 | 6.44 (0.77,12.12),0.03 |
| Small objects | Ref | Ref | Ref | Ref | Ref |
| Seizure frequency |  |  |  |  |  |
| Never or controlled | Ref | Ref | Ref | Ref | Ref |
| Monthly or less | -1.02 (-2.44,0.39),0.16 | 1.84 (0.86,2.82),<0.01 | -0.35 (-0.88,0.19),0.20 | -0.21 (-0.94,0.52),0.57 | 1.63 (-2.96,6.23),0.48 |
| Weekly | -0.23 (-1.99,1.54),0.80 | 2.17 (0.95,3.39),<0.01 | 0.20 (-0.46,0.87),0.55 | -0.08 (-0.99,0.82),0.85 | 4.55 (-1.18,10.27),0.12 |
| Daily | -0.28 (-2.25,1.68),0.78 | 1.54 (0.18,2.90),0.03 | 0.31 (-0.43,1.05),0.41 | -0.39 (-1.40,0.62),0.45 | 0.61 (-5.92,7.15),0.85 |
| Constipation |  |  |  |  |  |
| No | Ref | Ref | Ref | Ref | Ref |
| Yes | 0.55 (-0.75,1.84),0.41 | 0.61 (-0.33,1.54),0.21 | 0.31 (-0.18,0.80),0.21 | 0.61 (-0.05,1.26),0.07 | 3.60 (-0.62,7.82),0.09 |
| Reflux |  |  |  |  |  |
| No | Ref | Ref | Ref | Ref | Ref |
| Yes | -0.41 (-1.60,0.78),0.50 | -1.58 (-2.41,-0.74),<0.01 | -0.39 (-0.84,0.05),0.08 | -0.21 (-0.82,0.40),0.50 | -3.82 (-7.66,0.02),0.05 |
| Insomnia |  |  |  |  |  |
| Normal | Ref | Ref | Ref | Ref | Ref |
| Abnormal | 2.51 (1.21,3.81),<0.01 | 0.44 (-0.54,1.42),0.38 | 1.06 (0.58,1.55),<0.01 | 0.69 (0.01,1.38),0.05 | 7.55 (3.29,11.81),<0.01 |
| Excessive daytime sleepiness |  |  |  |  |  |
| Normal | Ref | Ref | Ref | Ref | Ref |
| Abnormal | 2.26 (0.80,3.72),<0.01 | 0.25 (-0.84,1.34),0.65 | 0.68 (0.12,1.24),0.02 | 1.20 (0.45,1.94),<0.01 | 8.08 (3.28,12.88),<0.01 |
| Data source |  |  |  |  |  |
| AussieRett | Ref | Ref | Ref | Ref | Ref |
| InterRett | -1.57 (-2.74,-0.40),0.01 | -0.74 (-1.60,0.12),0.09 | -0.55 (-0.99,-0.11),0.02 | -0.97 (-1.56,-0.38),<0.01 | -6.09 (-9.89,-2.30),<0.01 |

Ref, reference category; CI, confidence interval

**Additional File 1 Table 6. RSBQ scores (6-factor, Oberman et al., 2023) by covariates in 186 children with Rett syndrome**

|  | **Subscale** | | | | | | **Total***  (0-90) |
| --- | --- | --- | --- | --- | --- | --- | --- |
| Factor (range) | **Factor 1**  Emotional & Disruptive Behaviour  (0-24) | **Factor 2** Breathing Problems  (0-10) | **Factor 3**  Rocking & Hyporeactivity  (0-10) | **Factor 4**  Fear/Anxiety  (0-8) | **Factor 5**  Hand & Other Stereotypies  (0-12) | **Factor 6***  Facial Movements  (0-4) |  |
|  | mean (SD) | | | | | | |
| Overall | 7.7 (5.1) | 4.1 (3.1) | 4.0 (2.4) | 3.6 (2.0) | 4.6 (2.9) | 1.4 (1.2) | 36.3 (14.8) |
| Age, in years |  |  |  |  |  |  |  |
| 2-12 | 8.9 (5.0) | 4.3 (2.8) | 4.1 (2.6) | 3.7 (2.1) | 4.9 (2.7) | 1.6 (1.2) | 38.4 (14.6) |
| 13-17 | 6.5 (4.9) | 3.9 (3.3) | 4.0 (2.2) | 3.4 (1.9) | 4.4 (3.0) | 1.3 (1.2) | 34.3 (14.8) |
| Variant |  |  |  |  |  |  |  |
| C-terminal | 5.8 (4.1) | 3.7 (3.1) | 3.6 (2.4) | 3.3 (2.3) | 3.9 (2.4) | 1.3 (1.3) | 29.5 (14.2) |
| Early truncation | 7.0 (5.1) | 4.4 (3.3) | 3.5 (2.7) | 3.0 (2.3) | 4.5 (2.9) | 1.4 (1.5) | 34.6 (17.0) |
| Large deletion | 6.8 (5.3) | 3.1 (3.6) | 4.2 (2.2) | 3.5 (1.8) | 4.2 (2.8) | 1.2 (1.1) | 33.3 (15.9) |
| p.Arg106Trp | 11.0 (6.2) | 4.7 (3.0) | 4.4 (2.8) | 3.4 (1.5) | 4.7 (2.9) | 1.4 (0.8) | 42.1 (16.4) |
| p.Arg133Cys | 9.8 (5.3) | 4.2 (2.6) | 4.4 (2.6) | 3.4 (1.9) | 6.4 (3.1) | 1.4 (1.5) | 40.8 (14.4) |
| p.Thr158Met | 8.2 (5.1) | 4.4 (3.1) | 4.2 (2.3) | 3.5 (2.3) | 5.2 (2.7) | 1.7 (1.1) | 38.2 (14.2) |
| p.Arg168* | 7.4 (3.6) | 3.9 (3.2) | 4.3 (2.6) | 3.9 (2.2) | 4.3 (3.2) | 1.6 (1.3) | 36.4 (15.1) |
| p.Arg255* | 6.8 (5.3) | 5.5 (3.5) | 4.5 (2.9) | 4.0 (2.2) | 4.1 (2.9) | 1.5 (1.4) | 38.6 (15.6) |
| p.Arg270* | 8.1 (5.8) | 4.1 (3.1) | 4.4 (2.1) | 3.2 (1.9) | 5.0 (3.0) | 1.6 (1.3) | 38.2 (16.2) |
| p.Arg294* | 10.1 (6.0) | 4.1 (2.9) | 3.6 (1.3) | 3.6 (2.0) | 5.5 (2.8) | 1.2 (1.2) | 38.9 (14.2) |
| Arg306Cys | 8.3 (3.7) | 2.7 (2.3) | 2.6 (2.1) | 4.1 (2.1) | 2.8 (2.4) | 1.5 (1.2) | 31.9 (13.8) |
| Other | 7.1 (5.4) | 3.9 (2.4) | 4.5 (2.1) | 3.5 (1.8) | 4.5 (2.6) | 1.1 (1.1) | 35.3 (12.4) |
| Unknown | 8.1 (6.7) | 4.1 (2.8) | 3.4 (1.8) | 3.9 (1.9) | 6.3 (3.8) | 1.4 (0.8) | 39.4 (12.8) |
| Walking ability |  |  |  |  |  |  |  |
| Unable | 7.2 (4.7) | 3.8 (3.0) | 4.3 (2.5) | 3.4 (2.2) | 4.1 (2.5) | 1.5 (1.2) | 35.0 (14.6) |
| Assisted | 7.3 (5.5) | 4.5 (3.1) | 4.1 (2.7) | 4.1 (2.0) | 4.6 (3.2) | 1.4 (1.3) | 37.5 (16.2) |
| Independent | 8.2 (5.1) | 4.0 (3.0) | 3.7 (2.1) | 3.3 (1.9) | 5.1 (3.0) | 1.3 (1.2) | 36.2 (14.0) |
| Hand function |  |  |  |  |  |  |  |
| Unable | 6.6 (4.2) | 4.0 (2.8) | 3.9 (2.3) | 3.5 (2.1) | 4.4 (2.9) | 1.6 (1.1) | 35.7 (12.1) |
| Large objects | 8.2 (5.9) | 5.0 (3.1) | 4.3 (2.6) | 4.0 (2.0) | 5.0 (2.9) | 1.4 (1.2) | 40.3 (16.3) |
| Small objects | 8.1 (5.1) | 3.5 (3.1) | 4.0 (2.3) | 3.3 (2.0) | 4.5 (2.9) | 1.4 (1.3) | 33.9 (15.2) |
| Seizure frequency |  |  |  |  |  |  |  |
| Never or controlled | 7.8 (4.8) | 3.9 (3.2) | 3.6 (2.3) | 3.3 (2.0) | 4.7 (2.9) | 1.4 (1.3) | 34.9 (14.3) |
| Monthly or less | 7.1 (4.8) | 3.9 (3.1) | 3.8 (2.2) | 3.6 (2.0) | 4.5 (2.9) | 1.3 (1.2) | 35.2 (14.4) |
| Weekly | 8.1 (5.9) | 4.9 (2.4) | 4.4 (2.7) | 3.8 (2.0) | 4.9 (2.9) | 1.6 (1.2) | 38.9 (16.3) |
| Daily | 8.5 (5.7) | 4.3 (3.1) | 5.4 (2.3) | 4.0 (2.2) | 4.4 (3.0) | 1.5 (1.1) | 39.5 (15.4) |
| Constipation |  |  |  |  |  |  |  |
| No | 7.6 (5.1) | 4.5 (3.2) | 4.2 (2.3) | 3.7 (2.0) | 4.6 (2.9) | 1.5 (1.3) | 36.9 (14.8) |
| Yes | 8.1 (5.2) | 3.3 (2.7) | 3.8 (2.5) | 3.3 (2.0) | 4.5 (2.9) | 1.2 (1.1) | 35.1 (15.0) |
| Reflux |  |  |  |  |  |  |  |
| No | 8.1 (5.2) | 4.2 (3.1) | 4.2 (2.4) | 3.7 (2.0) | 5.1 (2.9) | 1.5 (1.2) | 38.0 (15.3) |
| Yes | 6.9 (4.9) | 3.8 (3.0) | 3.8 (2.3) | 3.2 (1.9) | 4.0 (2.7) | 1.3 (1.2) | 33.5 (13.6) |
| Insomnia |  |  |  |  |  |  |  |
| Normal | 6.6 (4.8) | 4.0 (3.0) | 3.7 (2.3) | 3.2 (2.0) | 4.4 (2.9) | 1.3 (1.2) | 33.9 (14.1) |
| Abnormal | 9.9 (5.0) | 4.3 (3.1) | 4.8 (2.4) | 4.2 (2.0) | 5.1 (2.8) | 1.6 (1.2) | 41.5 (15.0) |
| Excessive daytime sleepiness |  |  |  |  |  |  |  |
| Normal | 6.8 (4.8) | 3.7 (3.0) | 3.6 (2.3) | 3.1 (1.9) | 4.3 (2.9) | 1.2 (1.2) | 32.9 (14.1) |
| Abnormal | 9.9 (5.3) | 5.1 (3.0) | 5.2 (2.0) | 4.7 (1.9) | 5.3 (2.9) | 1.9 (1.2) | 45.0 (13.2) |
| Data source |  |  |  |  |  |  |  |
| AussieRett | 8.5 (4.6) | 3.7 (2.9) | 4.2 (2.2) | 3.6 (2.0) | 4.9 (2.6) | 1.5 (1.1) | 37.4 (14.0) |
| InterRett | 7.1 (5.4) | 4.4 (3.1) | 3.9 (2.5) | 3.6 (2.1) | 4.4 (3.1) | 1.3 (1.3) | 35.6 (15.4) |

* n=185

SD, standard deviation

**Additional File 1 Table 7. RSBQ scores (7-factor, Oberman et al., 2023) by covariates in 179 adults with Rett syndrome**

|  | **Subscale** | | | | | | |  |
| --- | --- | --- | --- | --- | --- | --- | --- | --- |
| Factor (range) | **Factor 1**  Emotional & Disruptive Behaviour  (0-22) | **Factor 2** Breathing Problems  (0-10) | **Factor 3**  Fear/Anxiety  (0-8) | **Factor 4**  Hand & Other Stereotypies  (0-10) | **Factor 5**  Social Interaction  (0-8) | **Factor 6**  Walking/  Standing & Rocking  (0-8) | **Factor 7**  Facial Movements  (0-4) | **Total**  (0-90) |
|  | mean (SD) | | | | | | | |
| Overall | 6.4 (5.0) | 3.5 (2.9) | 3.0 (2.0) | 6.5 (2.8) | 2.9 (1.9) | 2.7 (2.1) | 1.2 (1.2) | 33.4 (12.8) |
| Age, in years |  |  |  |  |  |  |  |  |
| 18-24 | 6.8 (5.0) | 3.6 (2.8) | 3.3 (2.1) | 6.6 (2.6) | 3.2 (2.0) | 2.7 (2.2) | 1.3 (1.2) | 34.6 (13.1) |
| 25+ | 6.1 (5.0) | 3.4 (2.9) | 2.8 (1.8) | 6.4 (2.9) | 2.7 (1.8) | 2.6 (1.9) | 1.2 (1.2) | 32.1 (12.4) |
| Variant |  |  |  |  |  |  |  |  |
| C-terminal | 5.9 (4.4) | 3.8 (3.1) | 3.2 (2.3) | 6.0 (3.2) | 3.2 (1.9) | 2.2 (2.1) | 1.6 (1.2) | 32.7 (11.3) |
| Early truncation | 5.2 (5.2) | 3.8 (3.4) | 1.9 (1.7) | 8.1 (1.5) | 2.7 (1.5) | 2.2 (1.6) | 1.0 (1.1) | 31.9 (9.5) |
| Large deletion | 7.5 (4.4) | 4.4 (2.7) | 2.0 (1.4) | 7.5 (2.7) | 3.3 (1.8) | 3.6 (1.4) | 1.5 (1.1) | 36.8 (9.0) |
| p.Arg106Trp | 4.5 (3.6) | 2.8 (2.9) | 2.6 (1.9) | 6.6 (3.0) | 3.0 (1.8) | 2.7 (2.2) | 0.9 (1.1) | 30.5 (11.4) |
| p.Arg133Cys | 4.7 (4.7) | 2.0 (2.7) | 3.1 (2.0) | 3.9 (2.3) | 2.1 (2.1) | 2.6 (1.3) | 1.1 (1.1) | 26.0 (13.1) |
| p.Thr158Met | 6.1 (5.0) | 3.5 (3.2) | 3.4 (2.2) | 7.3 (2.9) | 2.6 (1.9) | 3.1 (2.3) | 1.1 (1.1) | 32.7 (12.5) |
| p.Arg168* | 6.0 (4.6) | 3.6 (3.3) | 3.2 (2.3) | 7.5 (2.0) | 2.9 (2.1) | 1.8 (2.3) | 1.3 (1.2) | 34.6 (14.5) |
| p.Arg255* | 6.9 (6.6) | 3.4 (2.7) | 2.8 (1.9) | 6.6 (2.9) | 3.3 (2.3) | 2.6 (2.5) | 1.1 (1.2) | 33.9 (17.4) |
| p.Arg270* | 6.1 (4.9) | 3.4 (2.4) | 3.0 (1.7) | 6.8 (2.7) | 3.1 (2.1) | 2.1 (2.8) | 1.2 (1.6) | 33.2 (13.8) |
| p.Arg294* | 8.8 (4.9) | 2.6 (2.8) | 3.8 (1.6) | 6.0 (2.8) | 2.6 (2.1) | 2.5 (1.5) | 0.8 (0.9) | 34.3 (10.2) |
| Arg306Cys | 8.5 (5.1) | 3.2 (2.3) | 4.0 (2.4) | 6.7 (2.4) | 3.3 (2.3) | 3.9 (1.6) | 1.4 (1.0) | 38.4 (14.3) |
| Other | 7.1 (5.5) | 4.5 (2.3) | 2.8 (1.9) | 6.5 (2.3) | 2.9 (1.7) | 3.2 (2.1) | 1.6 (1.4) | 35.7 (10.8) |
| Unknown | 5.3 (4.0) | 3.8 (3.7) | 2.8 (1.6) | 4.8 (3.9) | 3.2 (1.9) | 2.3 (2.0) | 0.8 (1.3) | 31.0 (15.8) |
| Walking ability |  |  |  |  |  |  |  |  |
| Unable | 5.1 (4.2) | 3.0 (2.5) | 2.7 (1.9) | 5.9 (2.5) | 2.4 (1.7) | 1.1 (1.5) | 1.1 (1.0) | 27.9 (9.2) |
| Assisted | 7.7 (5.4) | 4.2 (3.0) | 3.5 (1.9) | 7.4 (2.5) | 3.5 (2.0) | 3.7 (2.0) | 1.5 (1.3) | 39.3 (12.9) |
| Independent | 6.2 (4.8) | 3.0 (2.9) | 2.7 (2.1) | 6.0 (3.1) | 2.8 (2.0) | 2.9 (1.8) | 1.2 (1.2) | 31.8 (13.1) |
| Hand function |  |  |  |  |  |  |  |  |
| Unable | 5.9 (5.2) | 4.0 (2.9) | 2.8 (1.8) | 7.3 (2.3) | 2.9 (2.0) | 2.3 (2.2) | 1.3 (1.2) | 33.5 (12.3) |
| Large objects | 6.3 (4.2) | 4.0 (3.1) | 3.3 (1.9) | 8.1 (2.4) | 3.4 (2.0) | 2.8 (2.3) | 1.7 (1.3) | 37.7 (13.1) |
| Small objects | 7.1 (5.0) | 2.7 (2.6) | 3.2 (2.2) | 5.0 (2.7) | 2.7 (1.8) | 3.0 (1.8) | 1.0 (1.1) | 31.5 (13.0) |
| Seizure frequency |  |  |  |  |  |  |  |  |
| Never or controlled | 6.7 (5.3) | 2.5 (2.6) | 3.1 (2.2) | 6.2 (2.9) | 2.7 (2.0) | 2.6 (2.0) | 1.1 (1.2) | 32.0 (13.7) |
| Monthly or less | 5.4 (4.5) | 4.3 (2.7) | 2.9 (1.8) | 7.0 (2.5) | 2.8 (1.8) | 2.8 (2.1) | 1.4 (1.1) | 33.6 (10.6) |
| Weekly | 6.7 (4.0) | 4.7 (3.1) | 3.0 (2.1) | 6.9 (2.6) | 3.8 (1.9) | 2.6 (2.5) | 1.5 (1.3) | 36.4 (14.0) |
| Daily | 6.8 (5.8) | 4.1 (3.1) | 2.7 (1.7) | 6.0 (2.6) | 3.0 (1.8) | 2.5 (1.6) | 1.1 (1.1) | 33.9 (11.2) |
| Constipation |  |  |  |  |  |  |  |  |
| No | 6.2 (5.0) | 3.3 (2.9) | 2.9 (1.9) | 6.4 (2.8) | 2.8 (1.9) | 2.7 (2.1) | 1.1 (1.1) | 32.3 (12.7) |
| Yes | 7.0 (5.0) | 3.9 (2.8) | 3.5 (2.3) | 6.8 (2.5) | 3.2 (2.0) | 2.7 (2.1) | 1.7 (1.2) | 36.0 (12.9) |
| Reflux |  |  |  |  |  |  |  |  |
| No | 6.8 (4.7) | 4.1 (2.9) | 3.1 (2.0) | 6.4 (2.7) | 3.1 (1.9) | 2.6 (2.1) | 1.4 (1.2) | 34.8 (12.4) |
| Yes | 6.0 (5.3) | 2.5 (2.6) | 2.9 (2.1) | 6.8 (2.8) | 2.7 (2.0) | 2.7 (2.0) | 1.1 (1.1) | 31.4 (13.0) |
| Insomnia |  |  |  |  |  |  |  |  |
| Normal | 5.5 (4.6) | 3.4 (2.8) | 2.9 (2.0) | 6.6 (2.7) | 2.8 (1.8) | 2.5 (2.0) | 1.2 (1.1) | 31.5 (12.0) |
| Abnormal | 9.1 (5.2) | 3.8 (3.0) | 3.5 (1.8) | 6.3 (2.9) | 3.3 (2.2) | 3.2 (2.3) | 1.4 (1.3) | 39.0 (13.6) |
| Excessive daytime sleepiness |  |  |  |  |  |  |  |  |
| Normal | 5.9 (4.7) | 3.4 (2.9) | 2.8 (1.9) | 6.4 (2.9) | 2.7 (1.9) | 2.6 (2.1) | 1.1 (1.1) | 31.8 (12.6) |
| Abnormal | 8.8 (5.3) | 3.7 (2.6) | 4.0 (2.1) | 7.0 (2.2) | 4.0 (1.8) | 3.0 (2.0) | 1.7 (1.3) | 40.1 (11.6) |
| Data source |  |  |  |  |  |  |  |  |
| AussieRett | 7.7 (5.0) | 3.9 (2.9) | 3.6 (2.2) | 6.4 (2.7) | 3.4 (1.9) | 3.2 (2.0) | 1.7 (1.2) | 37.0 (13.4) |
| InterRett | 5.6 (4.8) | 3.2 (2.8) | 2.6 (1.8) | 6.6 (2.8) | 2.6 (1.9) | 2.3 (2.0) | 1.0 (1.1) | 31.0 (11.9) |

SD, standard deviation
